# Transforming Fragile Hydrogel Chips Into Standardized Cartridges via Contact Line Pinning for Robust Microfluidics

**DOI:** 10.1002/smll.202506075

**Published:** 2026-01-09

**Authors:** Sin‐Yung Siu, Chiu‐Wing Chan, Yisu Wang, Langcheng Feng, Yichen Su, Yin Chen, Christopher K C LAI, Pingan Zhu, Kangning Ren

**Affiliations:** ^1^ Department of Chemistry Hong Kong Baptist University Hong Kong SAR China; ^2^ Translational Research and Development Center for Biomimetic Microfluidic Systems Tsinghua University Guangzhou China; ^3^ Department of Mathematics Hong Kong Baptist University Hong Kong SAR China; ^4^ School of Biomedical Engineering Guangzhou Medical University Guangzhou China; ^5^ Department of Microbiology Faculty of Medicine The Chinese University of Hong Kong Hong Kong SAR China; ^6^ Department of Mechanical Engineering City University of Hong Kong Hong Kong SAR China

**Keywords:** ASTs (antibiotic susceptibility tests), hybrid microfluidic chips, hydrogel devices, plug and play, POCT

## Abstract

Hydrogel‐based microfluidic chips have gained significant attention in various fields, including drug delivery, tissue engineering, and biosensing. However, the practical implementation of hydrogel chips often faces challenges related to connection methods due to the inherent fragility and wet properties of hydrogels, which limit their broader applications as testing platforms. In this context, we present a novel approach to transform hydrogel chips into standard cartridges that are low‐cost, convenient to use, and suitable for mass production, effectively addressing these challenges and enhancing usability. Our cartridge design utilizes a hydrogel–plastic bonding scheme to provide mechanical support to the chip and incorporates a contactless connection method for efficient, tubing‐free chip‐to‐world interfacing. This contactless connection method allows for a gap width tolerance between the hybrid chip and chip holder of 60–600 µm, while accommodating static fluid pressures of up to approximately 9.5 kPa. We demonstrate the applications of cartridges produced using this method in antibiotic susceptibility tests and 2D cell culture experiments. These results indicate that our method presents a viable and accessible alternative for hydrogel‐based microfluidic chips, enhancing their applicability across diverse biomedical applications.

## Graphical abstract

Hydrogel‐based microfluidic chips revolutionize drug delivery, tissue engineering, and biosensing, yet face challenges due to fragility and connection difficulty. This study introduces a novel cartridge design that enhances usability through a hydrogel–plastic hybrid design and a contactless connection method. This innovative approach promises greater applicability to 2D cell culture including antibiotic susceptibility tests and inviting further exploration.

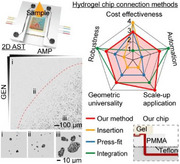

## 1 Introduction

Hydrogel has become a vital material in the construction of microfluidic chips due to its unique wet and hydrophilic properties, making it useful for applications in biomedical diagnostics, drug delivery, and tissue engineering. With its biphasic nature (liquid–solid) characterized by a high‐water content and a 3D porous polymer structure, hydrogel provides numerous benefits, including high permeability to small molecules, excellent gas permeability, biocompatibility, and biodegradability [1, 2]. These distinctive characteristics allow hydrogel to closely mimic the extracellular matrix (ECM) of cells, creating a microenvironment that closely resembles in vivo conditions [3]. Consequently, hydrogel itself has found applications across various fields of biological research; for instance, the disk diffusion test (known as agar diffusion test) is a well‐established and standardized method that employs hydrogels to determine bacterial susceptibility to antibiotics, which is expected to account for the largest share above 37.6% of the global antimicrobial susceptibility testing market in 2024 [4]. This phenotype culture‐based microbiological assay, commonly used in diagnostic and drug discovery laboratories, utilizes the physical properties of agar, such as its density and composition, to influence the rate and spread of antimicrobial diffusion. By combining hydrogel with microfluidics, hydrogel‐based microfluidic chips gain both perfusion and diffusion capabilities. This enables precise control of multiple biological parameters without being subject to the shear forces induced by fluid flow [5]. The exceptional properties of hydrogel‐based microfluidic chips have found wide applications in large‐scale tissue or organ models in vitro [6, 7], in vivo physiological and pathological simulations, drug screening, etc. [8, 9]. In contrast, conventional microfluidic chips made of rigid materials (such as plastic, glass, or polydimethylsiloxane (PDMS)) face inherent limitations in simulating the ECM environment [10]. The higher elastic modulus of these rigid materials (ranging from 360 kPa to 47.7 GPa) compared to the ECM (1–10 kPa) and their dense internal structures significantly restrict their permeability [11].

Ideally, technologies developed in the microfluidic device research field should be (commercially) mass‐produced and applied in areas that can benefit from their innovative capabilities. Microdevices made of plastics and paper have set good examples in large‐scale use [12, 13, 14]. However, hydrogel‐based microfluidic chips encounter significant challenges in becoming widely applicable devices, particularly for users outside the microfluidic research community. The fragile and soft nature of hydrogels complicates the assembly and manipulation of these microchips, making it difficult to standardize them for large‐scale use. Establishing a reliable chip‐to‐world fluid interface for hydrogel chips has been a long‐standing challenge; this interface is crucial in microfluidic devices as it connects the chip to the external macro‐scale environment. Achieving a dependable chip‐to‐world fluid interface enables fast connection and disconnection, allowing for convenient implementation and use by all users and researchers. Unfortunately, Dissimilar to conventional microfluidic chips, hydrogels cannot rely on self‐elasticity, holders, or adhesives for connection to tubing for fluid supply. Connecting hydrogel‐based microfluidic chips necessitates delicate manual operation, specialized chip design, and careful selection of hydrogel materials. Existing connection methods can be categorized as insertion [15, 16, 17], press‐fit [18, 19], and integration [20, 21, 22, 23, 24, 25] (Table S1). Although insertion and press‐fit are simple, they can easily damage the hydrogel or lead to channel collapse, particularly chips with high aspect ratio microchannels. To address these problems, some tough hydrogels have been developed by incorporating additives, such as poly(ethylene glycol), chitosan, or alginate. However, this solution also increases the elastic modulus of the chips (∼100–1000 kPa), making them incompatible with ECM as a result [26, 27]. Thus, integration methods have been developed to protect the hydrogel during operation, where the hydrogel is embedded into a conventional chip through filling [28, 29] or 3D bioprinting sacrificial microchannels [30], so‐called “gel‐in‐channel” or “gel‐in‐chamber”. These methods require elaborate designs or involve meticulous fabrication processes, emphasizing the technical expertise required to successfully integrate hydrogel materials into conventional chips. For example, in the filling approach, the chip requires micropillars or encapsulating membranes to prevent hydrogel leakage before solidification. This “gel‐in‐channel” or “gel‐in‐chamber” chip design restricts its functionality, such as the inability to mimic in vivo situations on the chip's surface (e.g., growth of a biofilm) [31, 32]. Alternatively, in 3D bioprinting, sacrificial microchannels are printed in a lure adapter with hydrogel building block support, adding complexity and difficulty to implementation. Consequently, current connection methods still fall short in providing an optimal solution for connecting hydrogel‐based microfluidic chips.

To develop a convenient and reliable method for establishing a chip‐to‐world interface compatible with various hydrogel types, we created a contactless interfacing scheme. This scheme enables secure and friction‐free connections between tubing and chips while providing mechanical support for the hydrogel chip at a low production cost. Although some contactless chip‐to‐world methods have been developed for plastic or PDMS microfluidic chips, relying on 3D T‐shaped inlet/outlet designs [33] or superhydrophobic coatings [34, 35, 36] to prevent leakage, they are not suitable for hydrogel chips. This is primarily because hydrogels are generally superhydrophilic, leading to fluid wetting the junctions instead of being pinned, even with specialized inlet/outlet designs, which results in leaks. Additionally, the inherently wet nature of hydrogels poses challenges for applying commercial superhydrophobic coatings. Consequently, these methods are ill‐suited for hydrogel‐based microfluidic chips. Furthermore, even if a superhydrophobic surface is created on the chip, its opaque nature would obstruct the use of microscopy imaging. Nonetheless, their contactless strategies inspired us to establish a promising concept of connecting the chips through contact line pinning.

Here, we present a contactless and leak‐proof hydrogel–plastic hybrid device for connecting hydrogel‐based microfluidic chips, utilizing surface tension contrast. Our device consisted of a disposable hydrogel–plastic hybrid chip and a reusable Teflon‐coated chip holder. The hybrid chip was fabricated by bonding or gluing a hydrogel to a plastic substrate to enhance its mechanical strength while preserving the hydrogel's intrinsic properties and optical transparency. To illustrate the versatility of this approach, two types of hydrogels, Agar‐poly(acrylamide*‐co‐N,N′*‐methylenebisacrylamide) (P(AAm*‐co‐*MBAA) hydrogel.) hydrogel and Agar‐genipin‐crosslinked gelatin (GN‐GEL) hydrogel, were utilized for chip fabrication in this study. Based on the properties of each hydrogel, these hybrid chips were applied for distinct applications separately. For the connection, the hybrid chip was inserted into the Teflon‐coated chip holder for automatic contactless connection and manipulation.

Our contactless connection strategy leverages the contact‐line pinning effect of hydrophobic Teflon coating, similar as rose petal effect, to effectively retain fluids within the liquid connection region of the gap. Unlike previously reported methods that rely on the Lotus effect, characterized by strong fluid repellence, our approach enables connections with hydrophilic chips even without the need of using 3D inlet/ outlet designs. Generally, the Lotus effect is most commonly adopted in the superhydrophobic surfaces for droplet repellency (roll‐off angle less than 10°) rather than strong pinning (roll‐off angle at 90°–180° ). In contrast, our method prioritizes this pinning effect to achieve leakproof connections. The rose petal effect facilitates precise liquid management and retention, distinguishing it from the Lotus effect, which primarily minimizes fluid contact. This makes our approach particularly suitable for applications requiring stable fluid connections in chip‐to‐world interfaces. Some contactless chip‐to‐world methods highlight the necessity of using a superhydrophobic coating with the same contact angle on both interfacial surfaces systematically pin the fluid. But when fluid flows between surfaces with different contact angles, the contact line moves freely, making it difficult to maintain a stable bridge across the gap. In contrast, our strategy leverages contact line pinning, which adapts to changes in wettability between surfaces with different contact angles (from 89° to 132°). This approach allows for unsystematic pinning, enabling a continuous flow of liquid across boundaries without leakage. By employing this strategy, our contactless chip‐to‐world method can effectively accommodate static fluid pressures of up to ∼9.5 kPa. Our strategy not only explores the new application of the pinning effect but also reduces production costs and enhances operational robustness.

Consequently, our method offers a universal platform for easily creating contactless chip‐to‐world interfaces for hydrogel microfluidic devices, transforming them into cartridges for efficient and standardized use. We also demonstrated the biological application of our device in both 1D and 2D antibiotic susceptibility tests (AST), and on‐gel cell culture, exhibiting its potential for biological and clinical applications.

## 2 Results and Discussion

### 2.1 Overall Design of Hydrogel–Plastic Hybrid Device

Although hydrogel hybrid devices are widely used and popular in microfluidics research, it is worth noting that most of them use elastomers as a substrate to graft hydrogels [37, 38]. This preference arises from the need to employ soft and elastic materials in common application scenarios such as soft robotics [39, 40], wearable sensors [41], and soft electronics [42]. However, elastomers have relatively low mechanical strength (∼3 MPa) [43] compared to other rigid materials, such as glass, metal, and plastic (2.9–407 GPa) [44, 45]. Consequently, employing a hard substrate for fabricating a hybrid chip can offer greater mechanical support for the hydrogel to prevent deformation during operation and allow for automatic connection. Also, the transparent and rigid substrate in a hybrid chip offers essential optical property for microscopic analysis in biological applications, while its rigidity and stability support the microchannels. This configuration enables high‐throughput screening, and continuous flow reactions, particularly at high flow rates, for example, drug screening and biomedical devices. Considering factors including transparency, biocompatibility, ease of fabrication, and cost, we selected polymethyl methacrylate (PMMA) as a chip substrate [46, 47, 48].

For the hydrogel part, hydrogels can be broadly classified into natural and synthetic materials. Two representatives, gelatin and P(AAm*‐co‐*MBAA) hydrogel, from each category were selected to illustrate the versatility of hybrid chip fabrication. Gelatin is a molecular derivative of collagen which is highly biocompatible and supports cell attachment due to its rich content of amino acids like glycine, proline, and hydroxyproline. However, its poor stability necessitates crosslinkers, such as genipin, to enhance their mechanical and thermal properties [49, 50]. Unlike gelatin, P(AAm*‐co‐*MBAA) hydrogel is a synthetic polymer known for its high strength and stability. Its properties can be easily tailored by adjusting polymer concentration, cross‐linking density, and functional groups, allowing for specific applications [51]. This ability to independently modulate stiffness and adhesive ligand presentation enhances our understanding of cellular responses, which is more difficult with natural materials [49, 52]. Therefore, considering its tailored properties, the P(AAm*‐co‐*MBAA) hydrogel was primarily used for general studies in this research.

**FIGURE 1:**
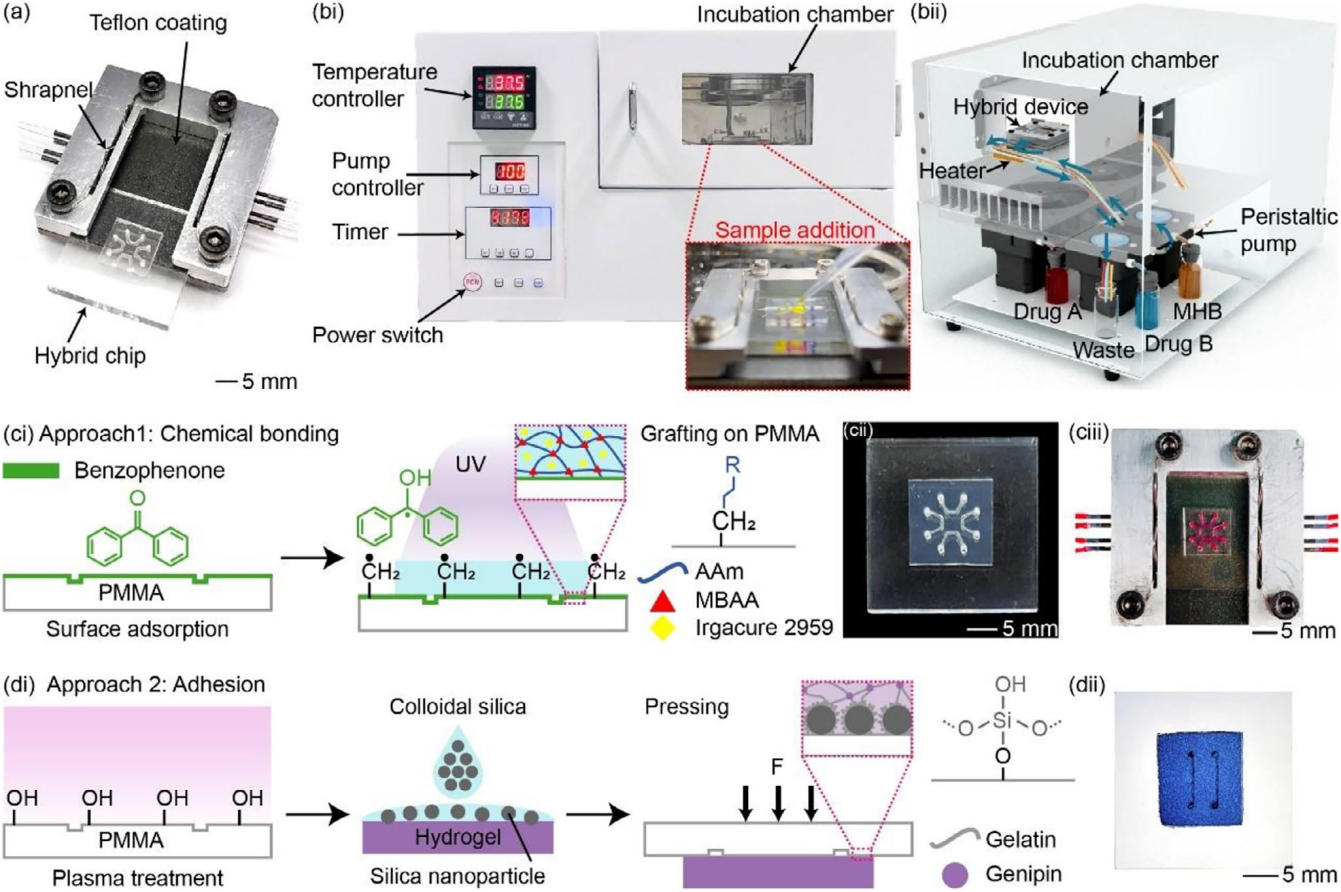
Overall design of our contactless connection approach for the hydrogel–plastic hybrid chip. (a) Image of our hydrogel–plastic hybrid device consists of a hybrid chip inserted directly into a Teflon‐coated chip holder for automatic connection. (b) Prototype of our AST automatic machine (left: front side (i), right: back side (ii)), along with a demonstration of its operational process (insert). Details are available in Figure S1. (c,d) Illustration of the two fabrication approaches for the hydrogel–plastic hybrid chip. (ci) Agar‐P(AAm*‐co‐*MBAA) hydrogel–plastic hybrid chip fabrication via chemical bonding. (cii) Images of the Agar‐P(AAm*‐co‐*MBAA) hydrogel–plastic hybrid chip. (cii) Image of Agar‐P(AAm*‐co‐*MBAA) hydrogel–plastic hybrid chip plastic hybrid device after connection and perfusion with red dye. (di) Agar‐GN‐GEL hydrogel–plastic hybrid chip fabrication via adhesion. (dii) Image of an Agar‐GN‐GEL hydrogel–plastic hybrid chip.

The hydrogel‐plastic hybrid device consisted of the hybrid chip and the Teflon‐coated chip holder (Figure 1a). The chip holder surface was coated with Teflon coating to increase its hydrophobicity. Also, the special geometry inlet/ outlet design of the chip holder facilitated the automatic alignment and connection of the hybrid chip upon insertion into the chip holder. In this way, the device is ready for use and enables automated operation without requiring specialized personnel, allowing it to work with our AST automatic machine for subsequent applications (Figures 1b, S1, and Video S1).

Our hybrid devices enable mass production at low cost by incorporating microchannels fabricated as open channels within a plastic substrate prior to bonding. Constructing microchannels on PMMA substrates, instead of hydrogels, provides the necessary mechanical strength for micromachining, allowing for mass production through various techniques such as computer numerical control (CNC), hot embossing, and laser cutting. To fabricate an Agar‐P(AAm*‐co‐*MBAA) hydrogel‐plastic hybrid chip (Figure 1c), the hydrogel was laid on the PMMA substrate and exposed UV light, which chemically crosslinked to form polyacrylamide and bonded the hydrogel to the PMMA substrate simultaneously. Notably, the double network structure formed by the physical crosslinking (agar component) and chemical crosslinking (P(AAm*‐co‐*MBAA) component) prevented any swelling issues in the hydrogel, which its elastic modulus is about 5.38 ± 0.09 kPa (Figure S2). The bonding mechanism, explained in SI Data 2 illustrated in Figures S3 and S4, relies on forming covalent bonds between amines and methyl groups [15, 38], enabling the bonding of different hydrogel types and substrates with compatible functional groups (e.g., polystyrene (PS), polypropylene (PP), and polyethylene (PE). This method offers users a universal and flexible approach to select P(AAm*‐co‐*MBAA) hydrogels with different additives or other UV‐cured hydrogels, and bond them onto diverse plastic substrates. As a result, our approach provides flexible material selection and is compatible with existing mass production techniques, with fabrication costs ranging from $0.82 to $22.84 per chip, primarily depending on the hydrogel and substrate fabrication methods. This agar‐P(AAm*‐co‐*MBAA) hydrogel–plastic hybrid chip was subsequently utilized for AST.

Alternatively, an adhesive method, such as hydrogel gluing [53], can be employed for hybrid chip fabrication as Figure 1d. A colloidal silica solution serves as the adhesive, bonding the Agar‐GN‐GEL hydrogel to the PMMA substrate. The silica nanoparticles anchored into the hydrogel network, interacting with the reactive hydroxyl groups on the PMMA to form hydrogen bonds, thereby achieving adhesion. The Agar‐GN‐GEL hydrogel, consisting of agar and genipin, enhanced thermal stability [50, 54] and prevented swelling, which its elastic modulus is about 5.38 ± 0.09 kPa (Figure S2), facilitating its use in later cell culture experiments.

Building on these bonding and adhesion methods, users can select from various hydrogels, both natural and synthetic, tailored to their specific needs in hybrid chip fabrication without compromising the inherent properties of the hydrogel. In addition, two key factors can be considered for hydrogel selection: first, the mechanical strength of the hydrogel must be adequate to create a stable microchannel, as insufficient strength could lead to channel collapse. Second, if the hybrid chip is intended for “cell‐on‐top” operation mode, which facilitates microscopy analysis or further treatment after experimentation, the chosen hydrogels must effectively support cell adhesion on their surfaces. This capability enhances single‐cell tracking and imaging, facilitates the identification and differentiation of bacterial species, and enables cell transfer for future analysis, all of which are vital for clinical microbial studies. While agar plates, commonly used as standard growth media for microorganisms (3.1–3.3 kPa), establish a guideline for the minimum mechanical requirement for applications with these specifications. Based on these factors, users can flexibly adjust the hydrogel's composition, crosslinking density, and polymer type to meet specific needs, such as tailored biochemical cues or the biophysical characteristics of the ECM.

### 2.2 Contactless Leak‐Proof Principle

**FIGURE 2:**
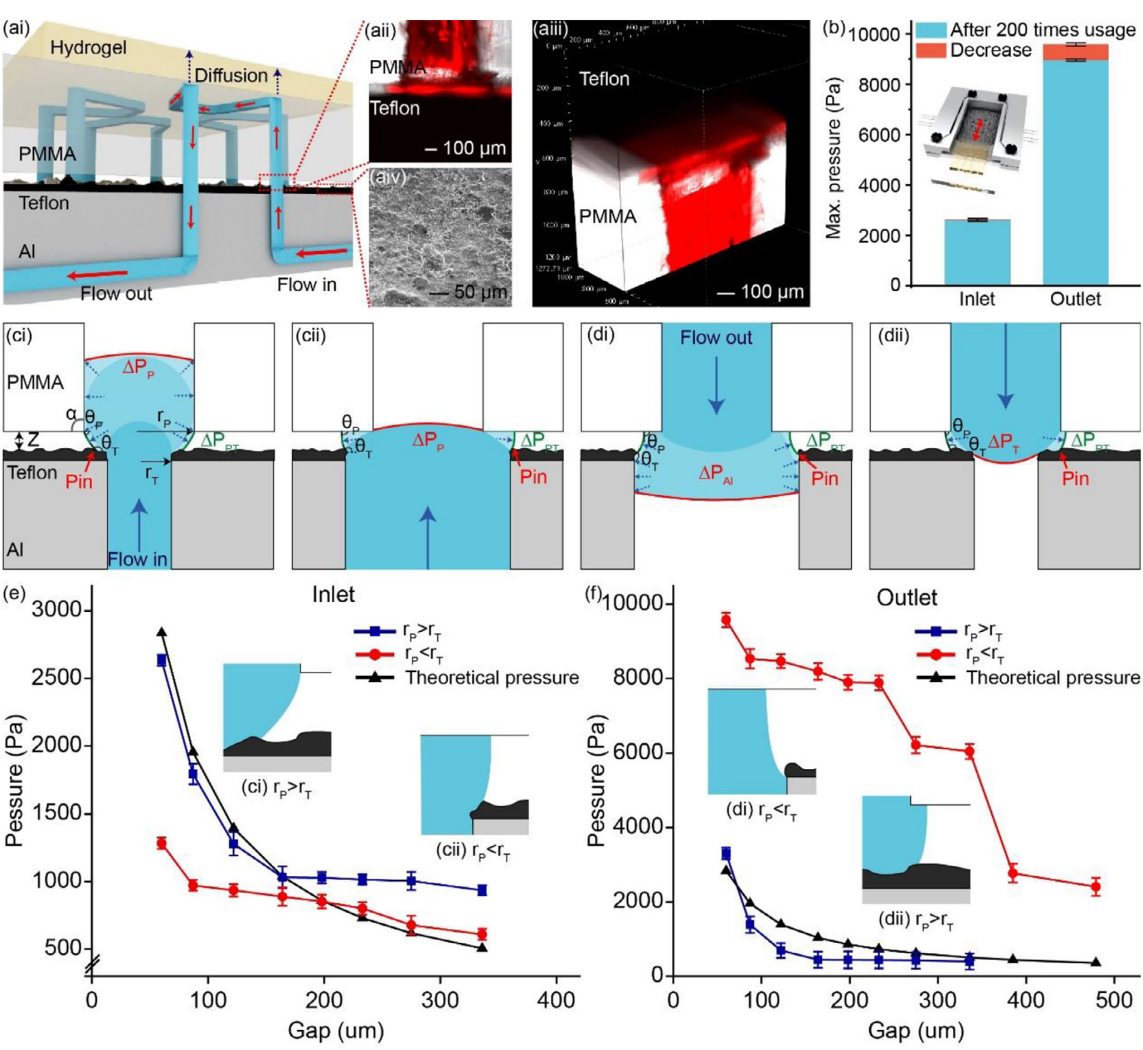
(a) Illustration of the flow direction of the hydrogel–plastic hybrid device after connection. The fluid flowed into the hybrid chip and diffused into the hydrogel. Due to the hydrophobicity and roughness of the Teflon coating, the fluid was pinned and formed a liquid bridge in the gap instead of leakage. The 2D (aii) and 3D (aiii) confocal images of the gap during perfusion were taken with the use of fluorescent dye (rhodamine B). (aiv) SEM image of rough Teflon coating. (b) Leakage pressure measured before and after 200 repeated uses of the chip holder. (c,d) Schematic drawing of the leak‐proof principle in two situations: the radius of the upstream was significantly larger than the downstream (i); and in the opposite case (ii), for both inlet (c) and outlet (d). (e,f) The measured statics pressure diagram of the inlet(e) and the outlet (f) under various gap distances.

Our hybrid device connected the hybrid chip to the holder based on the interfacial surface tension contrast between the PMMA substrate and the Teflon‐coated holder surface (Figure 2a). The rough texture of the Teflon coating (Figure 2aiv) hinders full contact between the chip and the holder (Figure 2aii,iii), creating a gap at the interface despite shrapnel compression, leading to trapped air forming a liquid–air interface and generating Laplace pressure to propel the fluid toward regions of lower pressure (inlet/ outlet) during perfusion. This design promotes preferential wetting, where the hydrophobic Teflon coating repels the fluid, enabling it to flow into the hydrophilic PMMA channels with wetting. This ensures a secure connection, even after 200 times of repeated use (Figure 2b).

According to Laplace's law, fluids tend to contract to minimize potential energy. Therefore, if the fluid could maintain a meniscus in the gap, indicating that cohesion dominated, we considered it stable and concluded that the chip had been successfully connected to the holder.

By comparing the Young–Laplace pressure (curvature pressure) between the inlet/outlet of the chip and the gap (interface of PMMA and Teflon), we can estimate whether the fluid is properly connected or if there is any leakage at the junction. In our assumptions, we considered that the radius of the inlet/outlet was either larger or smaller than the other (as drilling identical holes is not possible), the inner walls of all inlets and outlets were rough due to drilling, the Teflon coating was rough and had a fixed thickness, gravity effects were negligible, and the fluid used was water.

In general, the capillary pressure difference (∆*P*) is proportional to the contact angle according to the Young–Laplace equation, $\Delta P=-\frac{2\gamma \cos\theta }{r}$, where γ is the interfacial tension, θ is the contact angle and *r* is the channel radius. However, at the junction, the presence of dissimilar surfaces (Teflon and PMMA) caused the fluid to adopt an asymmetrically curved shape. Therefore, the surface tension of both materials needed to be considered. As a result, the capillary pressure difference was modified to $\Delta P_{\mathrm{PT}}=-\frac{4\gamma (\cos\theta_{P}+\;\cos\theta_{T})}{Z}$, where *Z* is the gap distance, subscript *P* represents PMMA, and *T* represents Teflon, *PT* represents the connection interface of PMMA and Teflon (measured contact angle data are shown in Figure S5 and Table S2). If Δ*P*_PT_ is larger than the ΔP of the inlet/outlet, it indicates a successful connection between the chip and the holder. Thus, achieving leak‐proof performance required Δ*P*_PT _> Δ*P*_P_ for the inlet and Δ*P*_*PT*_> Δ*P_T_* for the outlet. To prevent leakage, it is necessary to increase the difference between Δ*P_PT_* and Δ*P*. This could be achieved by reducing *Z* to increase Δ*P*_PT_ and by increasing *r* to reduce Δ*P*. In our study, we evaluated *Z*‐values ranging from 60 to 603 µm and *r*‐values from 372 to 961 µm. Our theoretical calculations indicate that when *Z* is minimized at 60 µm, the maximum Δ*P_PT_* achievable for leak‐proof is approximately 2.8 kPa. Conversely, when *Z* is maximized at 603 µm, Δ*P*_PT_ drops to a minimum of about 0.36 kPa, which is impractically low for most applications. The upper limit for the *Z*‐value was determined as $Z\;<\;2r(1+\frac{\cos\theta_{P}}{\cos\theta_{T}})$. The robustness of the connection was further examined through the tests of different *r‐*values alongside varying *Z‐*values.

When the fluid flows from the small *r* to the large *r* (*r*_T_ < *r*_P_ in the inlet; *r*_P _< *r*_T_ in the outlet), it will first reach the inner wall of the downstream channel and continue to spread until it reaches the open edge of the downstream channel (Figure 2ci, di). Once it reaches the open edge, the fluid will be pinned at the entrant edge, which has an open angle (*α*) of 90°. In the inlet, since PMMA is hydrophilic, the fluid is preferred to flow into the microchannel instead of leaking (Figure 2ci). In the outlet, due to the larger diameter of the outlet compared to the gap (372 vs. 60 µm), the fluid will first contact and wet the inner wall of the downstream channel rather than the hydrophobic Teflon coating surface (Figure 2di). Therefore, in the event of fluid leakage in the gap, it will need to traverse the Teflon coating vertically to reach the open edge first, with the traverse distance dependent on the thickness of the Teflon coating (∼20 µm). Following this, the fluid then needs to overcome the localized Young–Laplace pressures that arise on the rough Teflon surface in the horizontal direction. As a result, the fluid will be double pinned by the rough Teflon coating, leading to significantly higher leakage pressure in the outlet compared to the inlet (*r*_T _∼ 960 µm, outlet: ∼9.6 kPa > inlet: ∼2.6 kPa) (Figure 2e, f). Furthermore, due to the pinning effect caused by the Teflon surface and PMMA edge in the inlet (or PMMA surface and Teflon edge in the outlet), it allows for a larger *Z*.

On the contrary, when the fluid flows from the large r to the smaller r (*r*_T_ > *r*_P_ in the inlet; *r*_P_> *r*_T_ in the outlet), it reaches the surface of the downstream channel instead of the inner wall first. Consequently, the fluid will be pinned on the PMMA surface in the inlet (or Teflon surface in the outlet), and then expand outward until it reached its maximum contact angle (Figure 2cii, dii). As mentioned previously, for the fluid to enter the downstream channel without any leakage, it is necessary to have ΔP_PT _> ΔP_P_ in the inlet and Δ*P*_PT _> Δ*P*_T_ in the outlet. In this case, the connection relies on the surface tension contrast between the PMMA and the Teflon coating, as the fluid couldn't be double pinned by the edge of the downstream channel as in the above case. Hence, to prevent leakage by increasing Δ*P*_PT_, it is required to reduce Z, as mentioned earlier, which leads to a narrower acceptable range for Z. Again, it is more challenging for the fluid to cross the Teflon surface than the PMMA surface, resulting in the maximum leakage pressure in the outlet (∼3.3 kPa) being significantly higher than in the inlet (∼1.3 kPa). The resistance offered by the Teflon surface in the gap contributes to this notable difference in leakage pressures between the inlet and outlet.

To achieve a leak‐proof connection, a hydrophobic coating is essential on the interfaces. Instead of the typical approach of using hydrophobic coatings to repel fluids with low adhesion, this hydrophobic coating pins fluids with strong adhesion in designated areas, effectively preventing spillage. The experimental results suggest that allowing the fluid to flow from the upstream channel with a smaller radius to the downstream channel with a larger radius yields significantly higher maximum leakage pressure compared to the reverse case (inlet: ∼2.6 kPa vs. 1.3 kPa; outlet: ∼9.6 kPa vs. ∼3.3 kPa). This design not only ensures a robust connection with a wider range of Z, but also accommodates some degree of misalignment. It is because the upstream channel being consistently covered by the downstream channel. This design therefore reduces the manufacturing difficulty of maintaining gap distances and the complexity of precise microscale alignment. However, as the robustness of the connection is influenced by nonlinear 3D factors, further modeling is required for a comprehensive understanding of the phenomenon.

### 2.3 Characterization of Hydrogel‐Plastic Hybrid Chip

**FIGURE 3:**
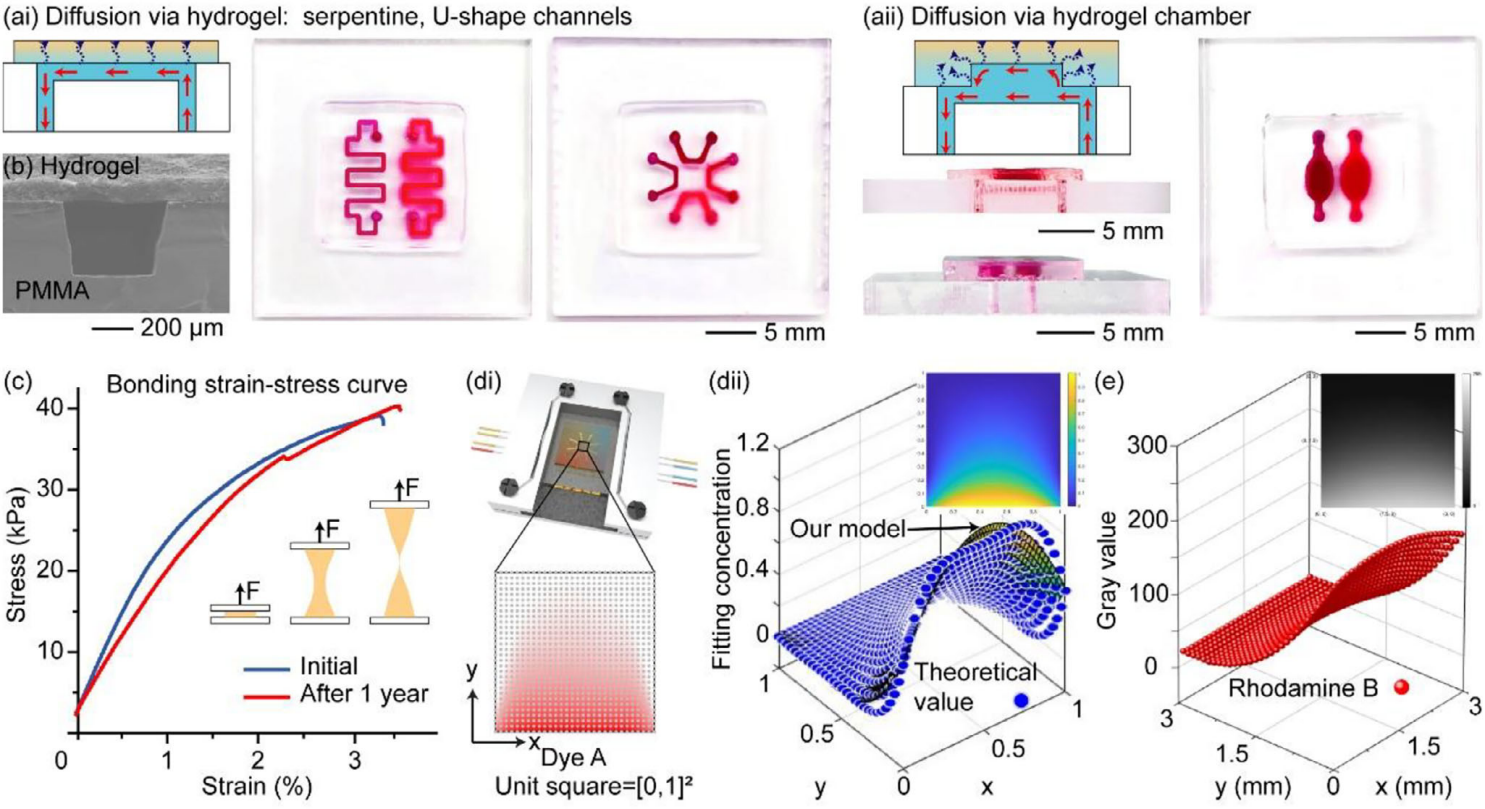
(a) Photos of Agar‐P(AAm*‐co‐*MBAA) hydrogel‐plastic hybrid chip with common designs include 2D channels (serpentine, U‐shaped) (i), and 3D hydrogel chambers (2D channels with hydrogel chambers) (ii). Red arrow: perfusion. Purple arrow: diffusion. (b) Cross‐sectional SEM image of Agar‐P(AAm*‐co‐*MBAA) hydrogel–plastic hybrid chip. (c) Strain–stress diagram of debonding Agar‐P(AAm*‐co‐*MBAA) hybrid chip before and after 1‐year storage. (d,e) 2D Diffusion on a hybrid chip. (d) Spatial domain illustration of diffusion in the hydrogel (i), and our numerical solution method (ii). (dii) Calculated diffusion concentrations derived from partial differential equations (PDE) solutions, with blue points indicating concentrations from Laplace's equation and the color map showing results from our mathematical model. Insert: the numerical simulation result of 2D diffusion starts with an initial concentration of 1 (yellow). (e) 2D Diffusion test results. Insert: the fluorescence image after stabilized at 120 min.

To ensure versatility for various application scenarios, it is crucial to have a hybrid chip design that exhibits high flexibility and robustness. In our study, we showcased three common chip designs: serpentine channels, U‐shape channels, and parallel channels with hydrogel chambers (Figure 3a). These designs effectively address the requirements for creating diffusion gradients, incubation, and mixing in a general context [18, 55, 56, 57, 58]. We demonstrated two diffusion scenarios: first, fluid is perfused into a microchannel and diffuses into a flat hydrogel (Figure 3ai); second, fluid is perfused into both a microchannel and a hydrogel chamber, diffusing into the hydrogel constructed with that chamber (Figure 3aii). This design allows for rapid cell loading without the need for valves/liquid exchange and meets low shear stress requirements to facilitate bacteria/cell attachment [59]. By constructing the microstructures on the PMMA substrate, we prevented issues like channel collapse or deformation that can occur on whole‐hydrogel microfluidic chips during bonding and operation (Figure 3b). Moreover, the morphology and geometry of microchannels could be precisely controlled in mass production. To assess the bonding strength of the Agar‐P(AAm*‐co‐*MBAA) hydrogel–plastic hybrid chip, we conducted a series of tests, which measured a debonding stress of approximately 40 kPa. This value remained stable (39 kPa) even after 1 year of storage (Figure 3c). In addition, the chip exhibited a prolonged shelf life and demonstrated the ability to recover after dehydration (Video S2). Furthermore, the chip's maximum flow rate reached approximately 3 mL/min (∼2.7 kPa), significantly surpassing the common flow rates observed in hydrogel‐based microfluidic chips (typically in µL/min). Thus, our hydrogel–plastic hybrid chip not only boasts an extended shelf life and cost‐effectiveness but also demonstrates comprehensive robustness, making it highly suitable for large‐scale applications in microfluidic systems.

The high stability of the hybrid chip allows for precise control over the morphology and geometry of the microchannels, enabling efficient diffusion processes. Unlike other microfluidic chips that rely on large reagent volumes for steady perfusion [60], our hybrid chip operates through a static diffusion process with minimal reagent consumption. To assess the diffusion performance of our hybrid chips, we conducted tests in both 1D and 2D scenarios. In the 1D scenario, a fluorescent dye diffused from a parallel channel (Figure S7a), while in the 2D scenario, two dyes diffused from two distinct U‐shaped channels (Figure S7b,c). The results indicated that the 1D scenario produced a linear gradient (Figure S7aii), while the 2D scenario resulted in a radial gradient (Figure S7bii, cii). This difference in patterns was attributed to the dye being diluted with both DI water and another dye from three distinct directions [61]. For the Agar‐P(AAm*‐co‐*MBAA) hydrogel–plastic hybrid chip, the concentration gradient was stabilized after 60 min in 1D (Figure S7ai) and after 120 min in 2D (Figure S7bi‐ci). For the Agar‐GN‐GEL hydrogel plastic hybrid chip, the diffusion stability test was extended to 3 days to assess long‐term cell culture usage, revealing that the concentration gradient stabilized after 60 min (Figure S12a). This stability highlights the hydrogel's potential for cell culture and proliferation studies, allowing researchers to effectively observe cell growth and differentiation over time. Additionally, it may facilitate drug testing and toxicity assays by enabling evaluations of chronic exposure, as well as microbial studies that monitor biofilm formation and responses to antibiotics [32].

To fully utilize the chip, the square area surrounded by the channels was designated as the analysis area, with the corners defined as the intersection points of the extended channel segments. Since the channels do not actually meet, the transition from 2D diffusion in the central region to 1D diffusion in the corner areas is not well‐defined. Also, when the chip design is changed, simply scaling the experimental results to obtain quantitative data may not accurately represent the actual situation. Therefore, we developed a mathematical model to study these complex situations. Based on our mathematical model, the simulation results aligned with our experimental results and were compatible with various designs (Figure 3d,e). This model could be flexibly adapted to calculate concentration distributions for different initial concentrations and diffusion domain sizes, accommodating a wide range of chip designs and experimental conditions (SI Data 5).

### 2.4 Rapid AST With Single‐Cell Imaging on an Agar‐P(Aam*‐co‐*MBAA) Hydrogel–Plastic Hybrid Chip

To assess the performance of our hybrid devices in microbial studies, we conducted AST experiments on an Agar‐P(AAm*‐co‐*MBAA) hydrogel–plastic hybrid chip to demonstrate its potential. As mentioned, our hybrid chips generate a concentration gradient through diffusion, allowing for direct addition of the bacterial sample onto the hydrogel for AST, rather than culturing in the microchannel. Wherefore, this cell‐on‐top strategy, combined with a highly transparent PMMA substrate, facilitates the monitoring of single‐cell growth and morphological changes under a microscope, even in bright‐field conditions. This approach enables phenotypic quantification for rapid AST through single‐cell image analysis. Currently, single‐cell imaging AST has been used not only to track individual cell development but also to reveal cellular heterogeneity, enable dynamic analysis, and uncover drug response variability, making it essential for biological research and promising for next‐generation analytical technologies [62, 63, 64].

**FIGURE 4:**
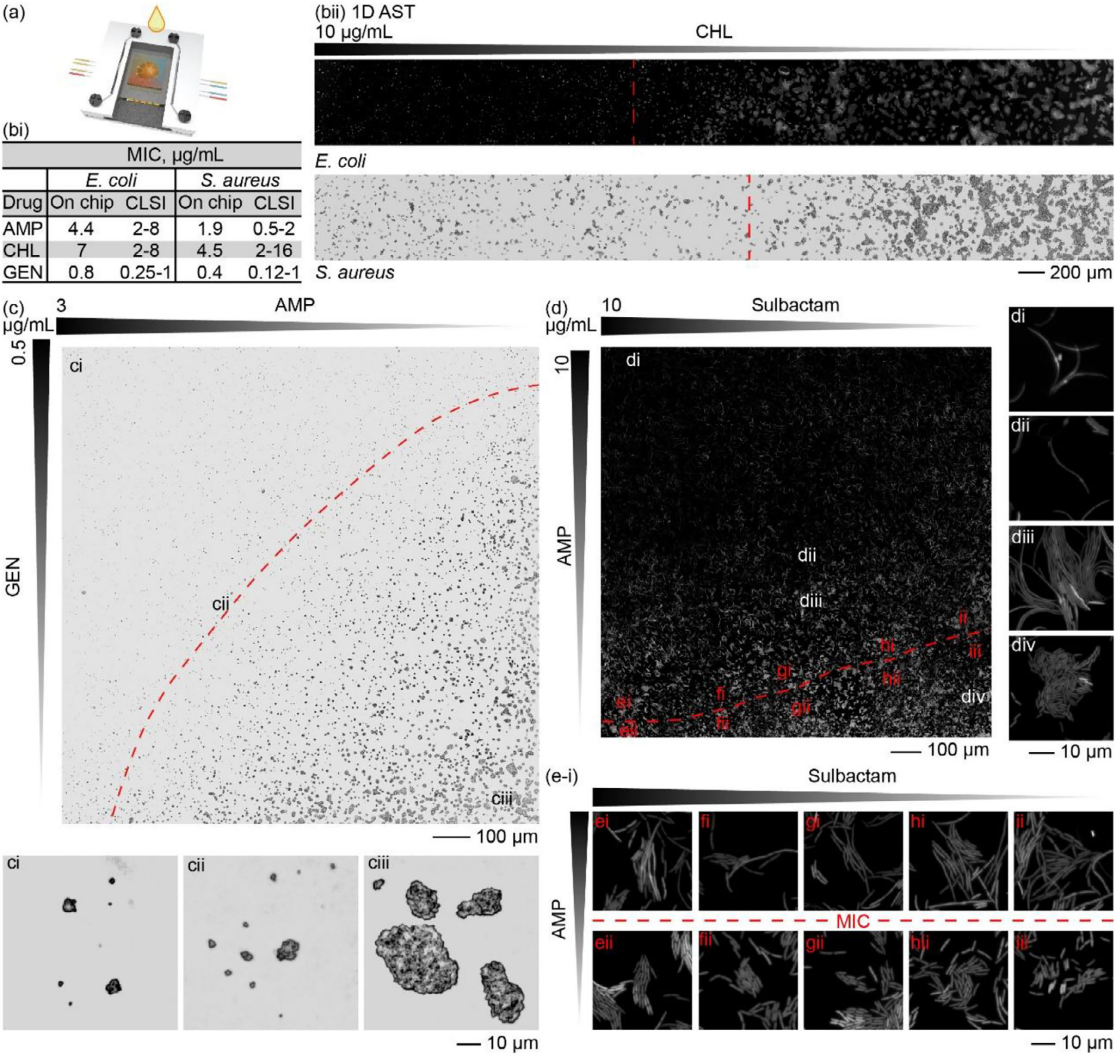
(a) Illustration of AST using an Agar‐P(AAm*‐co‐*MBAA) hydrogel–plastic hybrid chip. The bacteria sample was directly applied on the hybrid chip surface. (b) 1D AST results compared with CLSI standard (i) and selective result images (ii). MHB medium and a 10 µg/mL CHL antibiotic solution were separately perfused into parallel microchannels. The CHL solution diffused from left to right. (c,d) 2D AST results. (c) In the combined drug study with *S. aureus*, 0.5 µg/mL GEN diffused vertically, while 3 µg/mL ampicillin diffused horizontally. Changes in S. aureus quantity at various chip positions were measured (ci‐cii). (d) In the fixed‐dose combination study with GFP‐*E. coli*, 10 µg/mL AMP diffused vertically, while 10 µg/mL sulbactam diffused horizontally. Morphological changes of E. coli were assessed at different chip positions (di‐div). (e–i) Zoom‐in images show cell morphology changes above and below the MIC line. The red dotted line indicates the inhibition boundary (MIC line). All AST results images for *GFP‐E. coli* were recorded under fluorescence, while *S. aureus* results were captured under bright field.

To perform rapid AST, our hybrid chip was first immersed in Mueller Hinton Broth (MHB), a microbiological growth medium solution, ensuring a uniform concentration of MHB throughout both the medium channel and the hydrogel. This uniform distribution of MHB eliminated any disparities in bacterial growth during the test. Also, green fluorescence protein (GFP)‐expressing *Escherichia coli* (*E. coli*, ATCC 25922) and *Staphylococcus aureus* (*S. aureus*, ATCC 29213) were used as bacterial samples separately. After connecting with the chip holder, MHB medium and antibiotic solution(s) were perfused into the hybrid chip. For the 1D AST, the MHB medium and antibiotic solution were perfused into parallel microchannels separately as a 1D diffusion test, while for the 2D AST, the MHB medium and two different antibiotic solutions were perfused into adjacent microchannels separately as a 2D diffusion test. Once the chip generated a stable diffusion gradient of the drug(s), a drop (∼5 µL) of the bacterial sample was directly added on the hydrogel surface (Figure 4a). After 3–4 h incubation, the minimum inhibitory concentration (MIC) value of the antibiotic(s) was determined as the AST result. In this study, we employed three different classes of antibiotics with distinct mechanisms: ampicillin (AMP), chloramphenicol (CHL), and gentamicin (GEN), and along with a Beta‐lactamase inhibitor, sulbactam, for AST. These antibiotics are classified under penicillins, amphenicols, and aminoglycosides, respectively, according to the Clinical and Laboratory Standards Institute (CLSI) [65]. The AST results were evaluated by considering the mechanisms of action for each antibiotic, including growth inhibition or abnormal cell morphology change (e.g., elongation or burst). Then, the MIC values obtained were compared with the data from the CLSI for validation. In contrast to conventional AST microfluidics, our device offers the advantage of directly adding the bacterial sample onto the hydrogel surface instead of perfusing it into the channel [59, 60]. This cell‐on‐top strategy allows fast proliferation and colony formation, and eliminates the influence of shear flow and the issue of channel fouling [17, 66]. Therefore, our hybrid device offers high throughput and enables tracking of single cell morphology, facilitating faster analysis of drug effects on individual cells.

In contrast to traditional phenotypic AST methods such as disk diffusion, which measures the zone of inhibition to provide a range of MIC, and broth microdilution, which offers a MIC range based on a single doubling dilution endpoint, our device provides quantitative analysis by delivers exact MIC value along a continuous gradient. This is accomplished by diffusion rather than dilution creating a continuous drug concentration gradient, allowing the users to determine the MIC value at a specific drug concentration (Figure 4b). In the 1D AST, the MIC values for AMP, CHL, and GEN against GFP‐*E. coli* were determined as 4.4, 7, and 0.8 µg/mL under a fluorescent microscope (Figure S8). For *S. aureus*, the MIC values were found to be 1.9, 5.5, and 0.4 µg/mL for AMP, CHL, and GEN under the optical microscope, respectively (Figure S9). Notably, all these MIC values for the tested antibiotics were within the MIC range for ATCC strains, which were provided by CLSI [67] (Figure 4bi). These results not only affirm the reliability of our chips as tools for AST but also confirm that our approach retains the intrinsic properties of the hydrogel, including high permeability to small molecules, excellent gas permeability, and biocompatibility, as outlined in the introduction.

In 2D AST, checkerboard arrays and time‐kill assays are predominantly confined to research labs and have not been widely tested with a device for antibiotic combination testing [68]. Our device offers rapid AST in 2D, making it beneficial for clinical use. To validate our device in 2D AST, two different drug combinations were administered to GFP‐*E. coli* and *S. aureus* separately. First, we tested a synergistic effect of the AMP/GEN combination, which has been widely utilized and studied for its therapeutic benefits [60, 69]. The resulting inhibition boundary was observed to be roughly perpendicular to the diagonal line (Figure 4c), and the MIC values of AMP/GEN were found to be 1.4/0.1–0.4/0.6 µg/mL for GFP‐*E. coli* (Figure S11) and 0.46/0.43–1.9/0.12 µg/mL for *S. aureus*, respectively. The results showed that the MIC values obtained in the 2D AST were lower compared to the values from 1D AST, providing evidence that our hybrid device is suitable for investigating drug combinations and assessing their synergistic effects.

Second, we tested a fixed‐dose combination medication, AMP/sulbactam for GFP‐*E. coli*, which is a commonly prescribed beta‐lactam/beta‐lactamase‐inhibitor used to treat a wide range of infectious diseases including skin infections, pneumonia, urinary tract infections, and gynecologic infections [70]. Despite its weak antibacterial activity [71], sulbactam effectively enhances AMP's antibacterial activity by preventing the hydrolysis of AMP through binding to and inhibiting β‐lactamase enzymes [72, 73]. It is worth noting that the elongation effect of AMP on GFP‐*E. coli* can create an optical illusion [74, 75], causing the MIC line to seemingly move upward, which may result in an overestimation of the MIC value. Thus, to ascertain the true MIC line, the MIC value was determined when GFP‐*E. coli* exhibited abnormal cell length and no cell division [76, 77, 78]. The resulting inhibition boundary was observed as a red dot line (Figure 4d‐i), and the MIC value of AMP was found to be 2–3.4 µg/mL, which as lower than the MIC value obtained with AMP alone.

Since conventional phenotypic AST methods rely on assessing the turbidity of bacteria in the growth medium to determine the results, at least 24 h incubation is required for bacterial growth to reach appropriate concentrations (commonly a 0.5 McFarland standard) [67]. However, for certain bacterial infections, even a 6‐hour delay in treatment can have severe consequences, for example, the risk of death from sepsis increases by 7.6% per hour of delayed treatment [63]. Our method employs single‐cell analysis to confirm results at the single‐cell level, enabling rapid AST without the need for extended incubation time. As a result, our device can obtain AST results in just 3–4 h, even in 2D AST, thereby significantly expediting the diagnostic process for precision antibiotic selection. Moreover, our device has demonstrated its potential for various clinical applications, including but not limited to rapid AST.

### 2.5 On‐Gel Cell Culture on ‐Agar‐GN‐GEL Hydrogel–Plastic Hybrid Chip

To further assess the biological performance of our hybrid device in a cell culture study, we performed a cell culture test to demonstrate its potential. Since gelatin is extensively used for cell culture due to its non‐toxicity and ability to support cell attachment, we selected Agar‐GN‐GEL hydrogel for this experiment. Although a small amount of genipin was added to enhance the hydrogel's thermal stability, it is known to be a low cytotoxicity crosslinker that supported stem cell differentiation into neuronal progenitors [54].

Since our Agar‐GN‐GEL hydrogel–plastic hybrid chip supports a cell‐on‐top strategy, conducting experiments in on‐gel cell cultures facilitates clear insights and simplifies individual cell imaging and analysis. In this study, A549 cells were chosen as a model system due to their adhesion‐dependent growth behavior and their frequent use in microfluidic cell‐based assays for drug testing and lung disease modeling [79]. After three days of cell culture, the cells remained viable and continued to proliferate, with a cell viability rate of approximately 92.5% (Figure S12b). These results indicate that the Agar‐GN‐GEL hydrogel is non‐toxic to A549 cells and suitable for cell culture, underscoring the potential of our approach for cell studies within microfluidic systems.

Our Agar‐GN‐GEL hydrogel–plastic hybrid chip platform offers a well‐defined microenvironment for high‐resolution single‐cell imaging under controlled microfluidic conditions, providing a powerful tool for studying individual cell behaviors. Single‐cell imaging has enabled the creation of pedigree‐tree profiles, highlighting behavioral correlations among sister cells and their descendants, which can indicate cellular stress and inheritance responses to drug treatments [80, 81]. This capability is particularly useful for applications such as chemotaxis assays, where cell migration in response to a defined chemoattractant can be analyzed [82, 83]. Additionally, it facilitates studies on cell–cell interactions, lineage tracking, and phenotypic heterogeneity in cancer research, as well as immune cell dynamics and drug response profiling in personalized medicine [84].

## 3 Conclusion

Our innovative approach introduces a contactless method for enabling a chip‐to‐world interface for hydrogel‐based microfluidic chips. By harnessing surface tension contrast, this connection method demonstrates exceptional reliability, withstanding leakage pressures of up to approximately 9.5 kPa while allowing for a controlled gap between the chip and the chip holder. Unlike contactless conventional chip‐to‐world connections, our method eliminates the need for specialized 3D inlet/ outlet designs or superhydrophobic coatings on both interfaces, distinguishing it from existing technologies. This fundamental shift reduces fabrication complexity while preserving the optical properties of the chip substrate, which are crucial for high‐resolution imaging and analysis in biological applications. Compared to conventional contactless chip‐to‐world methods that rely on repelling fluids and often necessitate complex designs to avoid fluid contact, our method utilizes contact‐line pinning with simpler designs. This approach accommodates gaps and enables more precise management of fluid behavior within those gaps, eliminating the risk of leakage. Notably, our method is compatible with interfaces made up of two different wetting surfaces, hydrophilic and hydrophobic, unlike conventional methods that typically rely on two superhydrophobic surfaces. By ensuring more stable connections and enhancing operational robustness through strong adhesion, this pinning strategy allows fluids to be securely held in designated positions at the interface, even under varying flow rates. This significant advancement not only addresses tubing connection challenges but also enables automation, making it an ideal solution for a wide array of microfluidic applications with a maximum of 600 µm manufacturing tolerance for the chip‐holder connection. The versatility of our chip design enables customizable inlet and outlet sizes, ensuring robust connections while accommodating a degree of misalignment, as the upstream channel is consistently covered by the downstream channel. Additionally, our hybrid design accommodates various types of hydrogels and is compatible with multiple fabrication methods, including chemical bonding and gluing. For effective implementation, the hydrogel used in chip fabrication should possess sufficient mechanical strength to maintain the integrity of the microchannels over long‐term operations.

**FIGURE 5:**
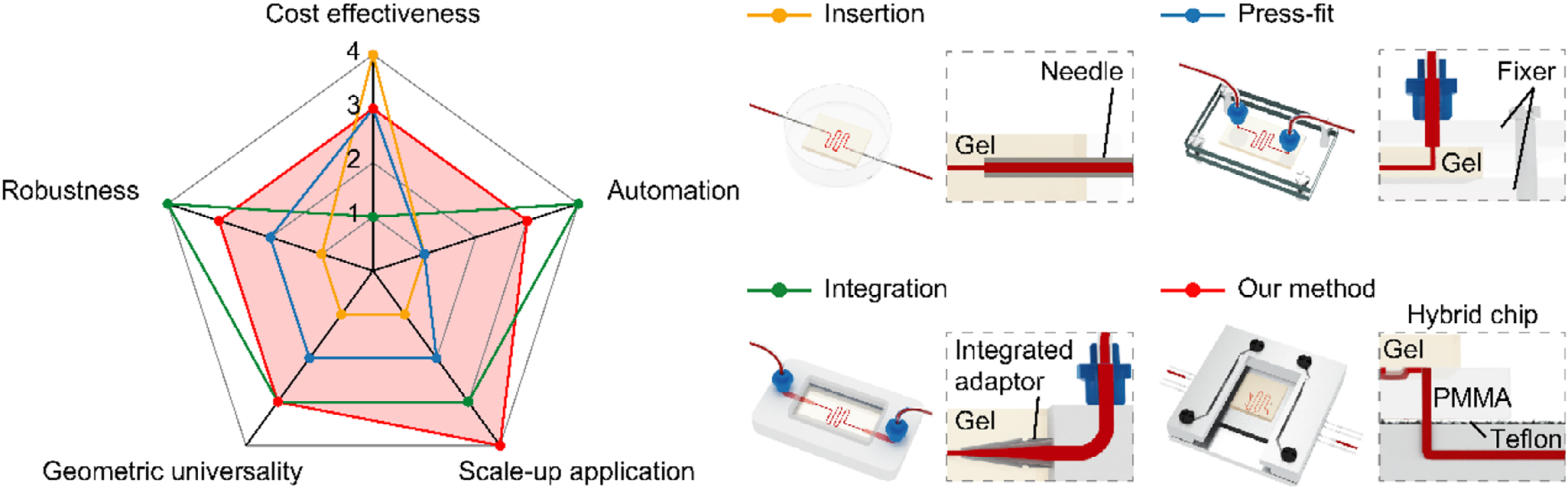
A radar chart comparing conventional hydrogel‐based microfluidic chip connection methods with our contactless method. Five key properties were assessed: cost‐effectiveness in overall fabrication, ease of automated operation, suitability for scale‐up application, universality in various inlet/outlet geometric designs, and connection robustness. (Ranking from 1 to 4, with 4 being the highest and 0 the lowest.).

Utilizing a cell‐on‐top strategy that allows single‐cell level analysis on drug gradients, our method guarantees rapid and quantitative AST results within just 3–4 h even for drug combination tests. Moreover, by employing a suitable hydrogel that promotes cell attachment, we successfully achieved on‐gel cell culture, showcasing its potential for cell study applications. This hybrid design not only offers immense promise for diverse areas such as tissue engineering, drug delivery, and biosensing but also features favorable biological properties and user‐friendly operation. By prioritizing simplicity, we eliminate the need for complex manufacturing processes or precise alignment, allowing for a controlled connection gap that facilitates mass production. The advantages of our connection method, in contrast to conventional methods for connecting hydrogel‐based microfluidic chips, are compared in a radar chart (Figure 5).

Ultimately, our strategy decisively overcomes the connection bottleneck of hydrogel‐based microfluidic chips and enables the production of hydrogel microfluidic cartridges, which liberates us from the constraints of precision manipulation and manufacturing, paving the way for automation and large‐scale clinical applications.

## 4 Methods

### 4.1 Materials and Equipment

LUDOX TM‐50 colloidal silica with 50 wt.% suspension in H_2_O, and porcine gelatin, type A, were purchased from Sigma‐Aldrich. *N, N′*‐methylenebisacrylamide (MBAA) was purchased from Tianjin Kemiou Chemical Reagent Co., Ltd., Tianjin, China. Benzophenone, and genipin were purchased from Shanghai Macklin Biochemical Technology Co., Ltd, China. Agar was purchased from AOBOX Biotechnology Co., Ltd., China. Irgacure 2959 and fluorescent dyes, including fluorescein and rhodamine B, were purchased from BASF, Germany.

Staphylococcus aureus (*S. aureus*, ATCC 29213) was purchased from Shanghai Fuxiang Biotechnology Co., Ltd., China. Green fluorescence protein (GFP)‐expressing Escherichia coli (*E. coli*, ATCC 25922) was obtained from Prof. Hongkai Wu from Hong Kong University of Science and Technology. Acrylamide (AAm), Mueller Hinton broth (MHB), antibiotics, including gentamicin (GEN) and chloramphenicol (CHL), and all other chemicals were purchased from Sigma‐Aldrich, US. Antibiotics, including ampicillin (AMP) and sulbactam were purchased from MedChemExpress, US. Polytetrafluoroethylene (Teflon) primer (Teflon 857G) and topcoat (Teflon 857G) were purchased from The Chemours Chemical Shanghai Co., Ltd, China. Polymethyl methacrylate (PMMA) and polystyrene (PS) plates were purchased from Shenzhen Meidian Acrylic Products Co., Ltd., China. The positive metal mold in the experiments was from Shenzhen Anbang Plastic Products Co., Ltd., China. The aluminum chip holder was purchased from Shenzhen Anyuxing Precision Technology Co., Ltd., China.

The homemade hot embosser was purchased by Shenzhen Xu Heng Tai Technology Co., Ltd., China. The field emission scanning microscopy (SEM) used in the experiment was from LEO 1530 with an energy dispersive X‐ray system from OXFORD. The confocal FLIM Imaging System used in the experiment was from Nikon C2si Plus. The fluorescence microscope used in the experiment was from Nikon, equipped with an Infinity 2 digital camera (Lumenera Corporation, Canada).

### 4.2 Hydrogel–Plastic Hybrid Chip Fabrication

Two types of hydrogels, gelatin and P(AAm*‐co‐*MBAA), were used for chip fabrication by employing different strategies. The plastic substrate was prepared either by CNC or by soft lithography. For soft lithography, the plastic substrate was placed on the positive metal mold in a hot embosser. The substrate was molded at a temperature higher than its melting point with slight pressure and then cooled down to room temperature. Notably, the pressure must be maintained throughout the whole thermo‐molding process.

### 4.3 Agar‐P(AAm*‐co‐*MBAA) Hydrogel–Plastic Hybrid Chip

Physical crosslinked AAm hydrogel was prepared by dissolving 2.3 g AAm, 5.1 mg MBAA, 20 mg Irgacure 2959, and 0.15 g agar into 10 mL DI water. The mixture was heated to a boil and stirred until completely dissolved. The gel solution was poured into a sterilized Petri dish and allowed to solidify. The solidified gel was cut to optimatethe optimate size for bonding.

The plastic substrate was cleaned by ethanol and water, then immersed into 10%w/v benzophenone‐ethanol solution for 10 mins. After adoption, the substrate was dried by nitrogen gas, and the physical crosslinked AAm hydrogel was placed on top of it. After exposure under UV for 5 mins, the hydrogel was chemically crosslinked to form P(AAm*‐co‐*MBAA) hydrogel and bonded on PMMA simultaneously. Finally, the hydrogel–plastic hybrid chip was immersed into DI water to remove all the unreacted reactants.

### 4.4 Agar‐GN‐GEL‐Plastic Hybrid Chip

Agar‐GN‐GEL hydrogel was prepared by dissolving 0.15 g of agar, 1.3 mm of genipin, and 0.48 g gelatin of in 10 mL of deionized water. The mixture was heated to boiling and stirred until fully dissolved. The gel solution was then poured into a sterilized Petri dish and allowed to solidify. After crosslinking, the hydrogel changed color from yellow to dark blue. The solidified gel was cut to an optimal size of 12 × 13 mm, and 10 µL of colloidal silica solution (Silica Ludox TM‐50) was evenly spread on its surface as an adhesive. The plastic substrate was modified by plasma treatment and then immediately pressed onto the hydrogel before the colloidal silica solution dried completely. Gluing was achieved by applying a contact pressure of 2.4 kPa for 3 h, allowing the colloidal silica solution to dry at the interface.

### 4.5 Teflon‐Coated Chip Holder

The bottom part of the holder was sprayed with the Teflon primer and heated at 150°C for 15 mins. Then, the Teflon topcoat sprayed on the holder and sintered at 350°C for 15 mins. The coating surface feature was characterized by SEM.

### 4.6 Connection Robustness Test

To study the service life of the chip holder with its connection reliability, the maximum leakage pressure measured before and after 200 repeated uses. Through‐holes were drilled in the PMMA and Teflon‐coated aluminum, matching the size of the chip and chip holder. Then, they were precisely aligned and pressed together under a microscope to establish the connection. Water flows from the Teflon‐coated aluminum to the PMMA substrate, designated as the inlet, while the reverse flow was designated as the outlet. The upstream channel was connected with the Teflon tubing and syringe barrel, and the downstream channel was blocked with adhesive tape. The syringe barrel was raised slowly until leakage occurred. Leak height was measured and used to calculate maximum leakage pressure. The gap distance was measured under a confocal microscope using rhodamine B. The diameter of the Teflon holes was found to be 372 and 961 µm separately. The diameter of the PMMA hole was found to be 594 µm. The gap distance was found to be 60 µm.

To investigate connection robustness across diverse chip designs and gap distances, through‐holes of varying diameters were drilled in both PMMA and Teflon‐coated aluminum for comparative analysis. Teflon spacers of different thicknesses were then inserted between the materials to create distinct gap distances. The maximum leakage pressure was calculated as previously mentioned. All the diameters of the drilled holes and the gap distances created by the Teflon spacers were measured under a microscope. The diameters of the holes were measured to be 372, 594 (original), and 961 µm, respectively. The gap distances were measured as follows: 60 (original: without space), 87, 122, 164, 198, 233, 275, 336, 385, 479, and 603 µm.

### 4.7 Strain–Stress Test

The Agar‐P(AAm‐*co‐*MBAA) hydrogel was bonded in between two PMMA plates and then immersed in DI water to remove all unreacted reactants. The bottom PMMA was fixed on the stage of a homemade material testing system, and the top PMMA was fixed with a fixture. The top PMMA was raised slowly, and the stress was measured by the system until debonding.

### 4.8 Characterization of the Hybrid Chip

The morphology of the channel was confirmed by SEM. The hybrid chip was dried at room temperature overnight before taking SEM. The flow resistance of the chip was characterized by injecting DI water into the chip at different flow rates until leakage occurred. The flow rate was maintained at 20 µL/min at the beginning and then increased in 20 µL/min intervals. After reaching 200 µL/min, the flow rate was increased in 200 µL/min intervals. The pressure difference inside the channel was calculated by using the Hagen–Poiseuille equation. For the recovery property, the hybrid chip was first immersed in ethanol until the hydrogel changed color from colorless to white for dehydration. Then, the chip was recovered by immersing it in DI water until the color of the hydrogel changed from white to colorless. The time needed for dehydration and recovery was recorded.

### 4.9 Diffusion Test

To evaluate the diffusion performance in both 1D and 2D scenarios, we conducted tests using two fluorescent dyes (fluorescein and rhodamine B), which have similar molecular weights to commonly used antibiotics. The dyes were dissolved into DI water at a concentration of 100 µg/mL. For the 1D diffusion test, the dye solution and DI water were injected into two parallel channels separately. For the 2D diffusion test, two types of dye solutions were injected separately into two adjacent channels; DI water was injected into the other two channels. The flow rate and temperature of the test were maintained at 0.25 mL/h and 37.5°C. Fluorescence images were taken by a fluorescence microscope every 30 min and analyzed by ImageJ 1.4 software.

### 4.10 Antibiotic Susceptibility Testing

For 1D AST, an antibiotic solution (diluted to the proper concentration in 2.1%w/v MHB) and MHB were injected separately into the channel at the same flow rate as described in the diffusion test. For 2D AST, antibiotic solutions were injected into two adjacent channels; MHB was injected into the other two channels. The bacterial sample was diluted to a suitable density (OD_600 _= 0.05), and then a drop of sample solution (5–6 µL) was applied and covered all channels on the chip. A sterilized Petri dish cap was used to cover the device and isolate it from the surroundings. The device was then incubated at 37.5°C. After completing the test, the resulting images were captured by a fluorescence microscope (for *E. coli*) or an optical microscope (for *S. aureus*) and analyzed by ImageJ 1.4 software. The exact concentrations of the antibiotics along the gradient on the chip could be accurately determined at each individual point, utilizing data from previous diffusion studies.

The MIC value or line was determined based on the working mechanism of the antibiotics. For AMP, one of the *β‐lactam* class antibiotics that function by inhibiting the bacterium cell wall synthesis, resulting in a weak cell structure and ultimately the lysis of bacteria [85]. Hence, the AST result was determined when the bacteria had the latter grow abnormally in length or growth inhibition. For example, under the effect of AMP, *E. coli* exhibit elongation, with a typical cell length ranging from 1 to 2 µm, serving as an indicator of inhibition [86]. For sulbactam, it was a *β‐lactamase* inhibitor that used with combination of *β‐lactam* class antibiotics to inhibit *β‐lactamase* [87]. For CHL, one of the amphenicols class antibiotics that function by blocking the enzyme peptidyl transferase on the 50S ribosome subunit of bacteria [88]. For GEN, one of the aminoglycosides class antibiotics that function by blocking the enzyme peptidyl transferase on the 30S ribosome subunit of bacteria contained as a portion of the molecule an amino‐modified glycoside [89]. Since CHL and GEN were both inhibit the protein synthesis, the AST result was determined when the bacteria had ceased their growth together.

### 4.11 A549 Cell Culture

A549 cells (human epithelial cell line derived from a lung carcinoma tissue, ATCC CCL‐185), were grown in a 90 mm cell culture dish in a total volume of cultured in 9 mL complete culture medium. They were kept at 37°C, 5% CO_2_ in humidified air in an incubator. At 80% confluence, cells were detached from the dish using 2 mL with 0.25% Trypsin solution (Thermo Fisher, U.S.), pelleted by centrifugation, and finally resuspended with fresh medium to the desired cell density.

The Agar‐GN‐GEL hydrogel was prepared in a 60 mm cell culture dish, followed by the application of 5 mL of phosphate buffered saline (PBS solution) and growth medium separately, allowing them to sit overnight. This process helped remove excess genipin and enabled the hydrogel to absorb the growth medium. Subsequently, A549 cells at a density of 0.8 × 10^6^ cells/mL were suspended on the hydrogel and cultured as previously described. Control A549 cells were cultured in a separate 60 mm dish under the same conditions. After 3 days of culture, cell viability was assessed using fluorescein diacetate (FDA)/propidium iodide (PI) staining. Fluorescence images were captured using a Confocal FLIM Imaging System (Nikon C2si Plus).

## Conflicts of Interest

The authors declare no conflict of interest.

## Supporting information




**Supporting File 1**: smll72190‐sup‐0001‐SuppMat.docx

**Supporting File 2**: smll72190‐sup‐0002‐VideoS1.mp4

**Supporting File 3**: smll72190‐sup‐0003‐VideoS2.mp4

## Data Availability

The data that support the findings of this study are available in the supplementary material of this article.
