# Supplementary material for: Transforming Fragile Hydrogel Chips Into Standardized Cartridges via Contact Line Pinning for Robust Microfluidics
Source: Small. 2026 Jan 9;22(11):e06075. doi: 10.1002/smll.202506075 (PMC12921542; doi:10.1002/smll.202506075)
Supplement: Supplementary file 1 — Supporting File 1: smll72190‐sup‐0001‐SuppMat.docx [file SMLL-22-e06075-s001.docx]

Supporting Information

**Transforming Fragile Hydrogel Chips into Standardized Cartridges via Contact Line Pinning for Robust Microfluidics**

*Sin-Yung Siu, Chiu-Wing Chan, Yisu Wang, Langcheng Feng, Yichen Su, Yin Chen, Christopher K C LAI, Pingan Zhu, and Kangning Ren^*^*

**1. Hydrogel-plastic hybrid automatic device**

An automatic incubation system was developed for AST as a demonstration for clinical application **(Figure S1)**, which is also appliable for another microfluidic test. This incubator can be divided into four main parts: drug storage, control panel, and operation chamber. First, the antibiotic solution and medium were stored in the side door before the use. After inserting the hybrid chip to complete the connection, the solutions were pumped into the chip holder by Peristaltic pump, and then perfused into the hybrid chip and further diffused into the hydrogel. The incubation temperature, flow rate, and testing time can be pre-set on the control panel. After generating the steady diffusion gradient, the bacteria sample solution can be added on the top of the hybrid chip inside the chamber. During the test, the state of the chip can be always observed through the window. After reaching the pre-set time, the chamber would be cooled down to room temperature by the cooling unit to stop the incubation immediately. Hence, the users can observe the results anytime and obtain more accurate results, especially for the bacteria sample that have a fast growth rate, e.g, *E. coli*. Finally, the users can take out the hybrid chip for further analysis.

**2. Fabrication mechanism of Agar-P(AAm-co-MBAA) hydrogel-plastic hybrid chip**

To fabricate an Agar-P(AAm-co-MBAA) hydrogel-plastic hybrid chip, the hydrogel was chemically bonded to the PMMA substrate while simultaneously undergoing chemical crosslinking through ultraviolet (UV) exposure. The PMMA surface was immersed in benzophenone solution for adsorption. Subsequently, an Agar- P(AAm-co-MBAA) hydrogel, which physically crosslinked by agar, was directly positioned on the open microchannels to form closed microchannels. Here, agar was used to P(AAm-co-MBAA) hydrogel swelling as well. Under UV exposure, the benzophenone adsorbed on the PMMA surface was exciting, generating benzophenone ketyl radicals by abstracting hydrogen from unreactive C-H bonds in PMMA. These radicals facilitate the grafting of polymer networks from the hydrogel onto the reactive sites on the surface. Simultaneously, the type I photoinitiator, Irgacure 2959, was activated to produce free radicals, initiating a polymerization reaction that forms poly(acrylamide). The bonding mechanism was explained in **Figure S2**. As a result, the hydrogel was chemically crosslinked and bonded on the PMMA substrate simultaneously, to form an Agar-P(AAm-co-MBAA) hydrogel-plastic hybrid chip as illustrated in **Figure S3**. The chip was finally immersed in deionized (DI) water for 30 minutes to remove any unreacted reactants through diffusion.

**3. Fabrication mechanism of Agar-GN-GEL hydrogel-plastic hybrid chip**

To fabricate an Agar-GN-GEL hydrogel-plastic hybrid chip, the hydrogel was adhered onto the PMMA substrate using a colloidal silica solution. The Agar-GN-GEL hydrogel was composed of agar and genipin, helping prevent swelling and increase thermal stability.^[1]^ When genipin is introduced to an agar-gelatin solution, it interacts with the amine groups of gelatin to form stable imine bonds, resulting in chemical crosslinking that produces a gelatin hydrogel. Then, the colloidal silica solution was applied directly to the Agar-GN-GEL hydrogel to let it absorb the silica nanoparticles. At the same time, the PMMA surface was activated by plasma treatment. When the hydrogel and PMMA were pressed together with force, the silica nanoparticles absorbed on hydrogel surface were further anchored in its network, and interacted with the reactive hydroxyl groups on the PMMA, forming hydrogen bonds that resulted in adhesion.

**4. Diffusion test**

In the 1D diffusion test, fluorescein and DI water were injected into parallel channels, and undergo the diffusion across the hydrogel surface. In the 2D diffusion test, fluorescein and rhodamine B were injected into an adjacent channel and DI water was injected into another two channels. The diffusion stability was found at ~1 hour for 1D diffusion and at ~2.5 hours for 2D diffusion **(Figure S7)**. The results showed that a stable gradient with good linearity was formed between the two channels in 1D diffusion. But in 2D diffusion, the dye was diluted with the DI water and another dye from other three directions (Liu et al., 2018).^[2]^ Hence, a radial shape concentration gradient profile was generated on the hydrogel in 2D diffusion. Hence, in 2D AST, the drug concentration at each position cannot be directly calculated as 1D.

**5. Mathematical model of 2D diffusion**

To simulate the 2D distribution of drug concentration within a mathematical model, our investigation focuses exclusively on the diffusion behaviour of drug A, assuming that drug B follows the same diffusion pattern **(Figure S9)**. In order to establish a general model, we initially assume a uniform concentration of one in the bottom channel, while maintaining a concentration of zero in the remaining three channels. Leveraging the widely recognized applicability of the Laplace equation, $\nabla^{2}u=0$,in describing diffusion processes, our objective is to employ its solution to estimate the concentrations of the drugs under consideration. By utilizing the solution of the Laplace equation, which characterizes the distribution and behaviour of a scalar field, we can approximate the concentration profiles of the drugs in this situation.

In our mathematical model, we consider the diffusion domain as a unit square [0,1]^2^ and establish a Cartesian coordinate system with x and y axes, setting the lower left corner as the origin. Within this unit square domain, we solve the Laplace equation to describe the diffusion process. However, it is important to note that the four corners of the unit square are not present or involved in the actual chemical model analysis. To ensure the uniqueness of the solution, we incorporate boundary information into our mathematical model based on experimental data. Specifically, we set the concentrations on the left, top, and right boundaries to zero, while the concentration on the bottom boundary is set to one. This allows us to treat the diffusion process as a 2D Laplace equation with Dirichlet boundary conditions.

To obtain a numerical solution, we employ the Meshfree method, specifically the Radial Basis Functions (RBFs) collocation method, in MATLAB to solve the Laplace equation and calculate the concentration values at each location within the unit square domain. Within the domain [0,1]^2^, we distribute 900 equally spaced points for spatial discretization. For the kernel function, we choose the Gaussian RBF function $\varphi(r) = e^{{-(\varepsilon r)}^{2}}$, where r represents the distance between points. To construct the collocation equations, we evaluate the Laplace equation at each collocation point $(x, y)$ within the domain. Using the RBF approximation, we express the solution $u$ as a linear combination of RBFs.

$$u(x, y) = \Sigmaᵢ \lambdaᵢ\varphi(||(x, y) - (xᵢ, yᵢ)||)$$

Here, $\lambdaᵢ$ represents the coefficients associated with the RBFs. The double vertical bars denote the Euclidean distance between the collocation point $(xᵢ, yᵢ)$ and the point $(x, y)$ where we want to evaluate the solution.

We also include a polynomial fit to the numerical RBF solution for easy comparison. Through this process, we obtain an estimated function as $f\left( x,y \right)=p00+p10*x+p01*y+p20*x^{2}+p11*x*y+p02*y^{2}+p21*x^{2}*y+p12*x*y^{2}+p03*y^{3}$.

Coefficients (with 95% confidence bounds):

p00= 0.469 (0.457, 0.481)

p10= 2.532 (2.487, 2.576)

p01= -2.333 (-2.399, -2.268)

p20= -2.54 (-2.582, -2.498)

p11= -2.725 (-2.827, -2.622)

p02= 3.373 (3.244, 3.502)

p21= 2.75 (2.678, 2.821)

p12= -0.0187 (-0.09006, 0.05265)

p03= -1.489 (-1.57, -1.407)

The generality of this model enables its application to various scenarios. For example, by considering the diffusion domain as a unit square, the model allows for scalability when applied to domains of different sizes. To estimate the drug concentration within the diffusion square domain [a, b]^2^, we initiate the process by scaling and shifting the domain to the unit square [0,1]^2^ through a coordinate transformation. This transformation involves calculating a scaling factor, denoted as $s= 1 / (b - a)$, for both the x and y coordinates. Next, we apply the coordinate transformation to map a point $(x, y)$ from the original domain to $(u, v)$ within the unit square. This is achieved by setting $m = \left( x - a \right)* s$ and $n = (y - a) * s$. After obtaining the transformed coordinates $(m,n)$, we substitute them into the function $u\left( x, y \right)$ to estimate the drug concentration. The function $u\left( x, y \right)$represents the mathematical model or equation used to approximate the drug concentration at a specific location in the original domain. By following this process, we successfully map the diffusion domain to the unit square, enabling us to estimate the drug concentration using the transformed coordinates $(m, n)$ within the function $u\left( x, y \right)$.

The model assumes an initial drug concentration of one, however, it can be flexibly adapted to estimate concentration distributions for various drug concentrations. By appropriately scaling the initial diffusion concentration, the model enables effective estimation of the concentration distribution across the entire hydrogel, accommodating a diverse range of scenarios characterized by varying drug concentrations.

**Table S1. Comparison of current connection methods for hydrogel chips.**

| Connection methods | Special requirement(s) | Cost^^^  (USD per chip) | Automatic operation | Scale-up application | Inlet/outlet geometric universality | Connection robustness |
| --- | --- | --- | --- | --- | --- | --- |
| Insertion^[3,4]^  -Directly insert the assemblies or connector into the hydrogel chip  e.g., needles, soft tube, or Luer adapter | UV glue or biological glue may be needed to steady the junction | $ 0.0028-19.2^[5]^  (Depending on the hydrogel and fabrication methods)  *With additional manual operation cost and waste expense from operational errors during connection | No, require trained personnel to avoid damaging the chips during insertion | No, labor intensive work is time consuming and limited throughput | No, close inlet/outlet intervals may cause debonding or deformation | Weak, failing under external forces  (Handling a maximum of ~3 kPa,^[6]^ depending on the bonding strength and hydrogel strength) |
| Press-fit^[7,8]^  -Press-fit the chip between a tailor-made plastic fixture, for further connection | Require tailor-made fixture; high aspect ratio channel may collapse/ deform | $ 0.0028-19.2^[5]^  (Depending on the hydrogel and fabrication methods)  *With additional cost for tailor-made fixture ($7-112, depending on the design)^[9,10]^ | No, require trained personnel for later encapsulation | No, while fixtures enhance operational efficiency, manual encapsulation is time consuming and limited throughput | Partially, spacing limited by the size of the connecting component employed in the fixture, e.g., needles, O-rings (diameter: ≥0.7mm)^[11]^, or Luer adapters (Outside diameter: 0.439-1.27cm)^[12,13]^ | Medium, fixtures can protect the chip from external force  (Depending on the hydrogel mechanical strength) |
| Integration  -Filling a hydrogel solution into central channel of a conventional chip, and then connected as traditional methods^[14–21]^ | Traditional chips need to have micropillar or membrane to block the hydrogel solution during filling | $220-614.20^[22,23]^  (Depending on the chip design) | Yes, chip housing provides enough strength for autoconnection | Yes, but fresh preparation of hydrogel inside the chip reduces efficiency, and the complex design of unreusable chip housing increases costs of use | Good, similar as conventional microfluidic chips | Excellent  (Depending on the mechanical strength of the traditional chip) |
| Integration  - Directly 3D print hydrogel sacrificial channel on a connector with Luer adapter, and then connected as traditional methods^[6,24]^ | Required 3D printing; limited to 3D printable hydrogel; sacrificial channel needs to be washed out after chip formation | $42.3- 526.50^[24–27]^  (Depending on the chip design) |  |  |  |  |
| Our method  -Push the hybrid chip into the Teflon coated holder for the connection | Universal and reusable Teflon coated holder | $ 0.82-22.84  (Depending on the hydrogel and substrate fabrication methods) | Yes, the PMMA substrate provides enough strength for automation, and the chip is autoconnected by contact line pinning after manual plugin | Yes, the Teflon coated holder is universal and reusable; the hybrid chip is cheap and easy to fabricate; labor-free connection strategy |  | Good, maximum pressure ~9.5 kPa (Depending on the surface tension contrast, radius of the inlet/outlet, and the gap distance) |

^ Assume that a total of 2.5 mL of hydrogel solution is used for chip fabrication.

**Table S2. Comparison of current connection methods for contactless connection methods for conventional chips.**

| Contactless connection methods | Description | Special requirement(s) | Mass production | Connection robustness |
| --- | --- | --- | --- | --- |
| Reentrant design^[28]^ | Using a re-entrant design (T-shape) at the inlet/outlet to hold the fluid by the surface tension | The inlet and outlet of the two vertically suspended microfluidic channels must be made in a T-shape on the microscale | No, fabricating the inlet/outlet in a T-shape on the microscale is difficult and costly | ~1.4 kPa  (Depending on the gap distance) |
| Parallel superhydrophobic interfaces^[29–31]^ | Coating superhydrophobic coating on both sides of the interconnecting surfaces to form a liquid bridge at the junction | Superhydrophobic coating needs to be coated on the disposable chip every time; V-groove on motherboard is needed for the alignment and maintaining the gap | No, disposable coated chips increase production costs; motherboards with V-groves increase the cost of changing chip designs | ~10-25.6 kPa  (Depending on the gap distance) |
| Our method | Push the hybrid into the Teflon coated holder for the connection | Universal and reusable Teflon coated holder | Yes, the Teflon coated holder is universal and reusable; the hybrid chip is cheap and easy to fabricate; labor-free connection strategy | ~9.5 kPa  (Depending on the surface tension contrast, radius of the inlet/outlet, and the gap distance) |


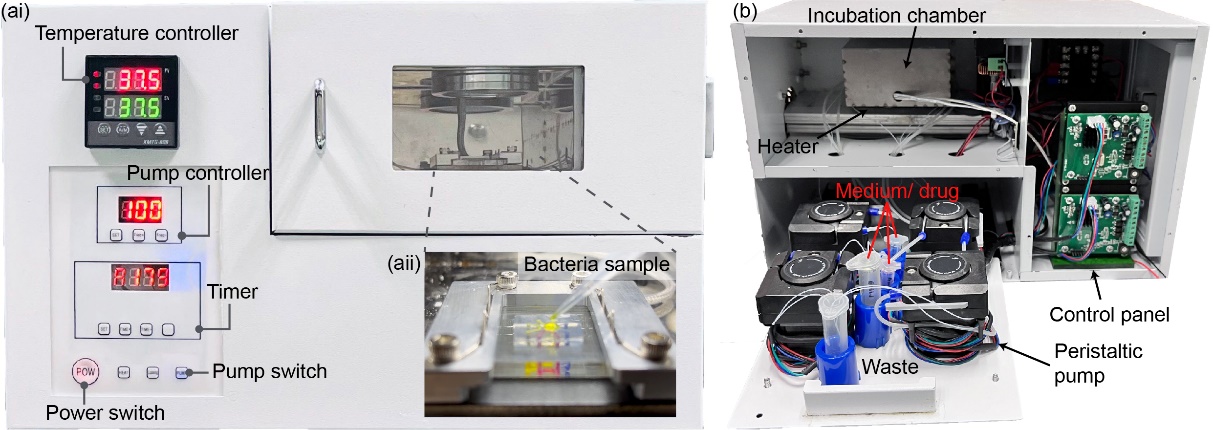


**Figure S1.** Illustration of automatic incubation system. (ai) Front side image of the incubator. (ai) Demonstration of adding bacteria sample in incubation chamber. (b) Back side scheme of the incubator.

**
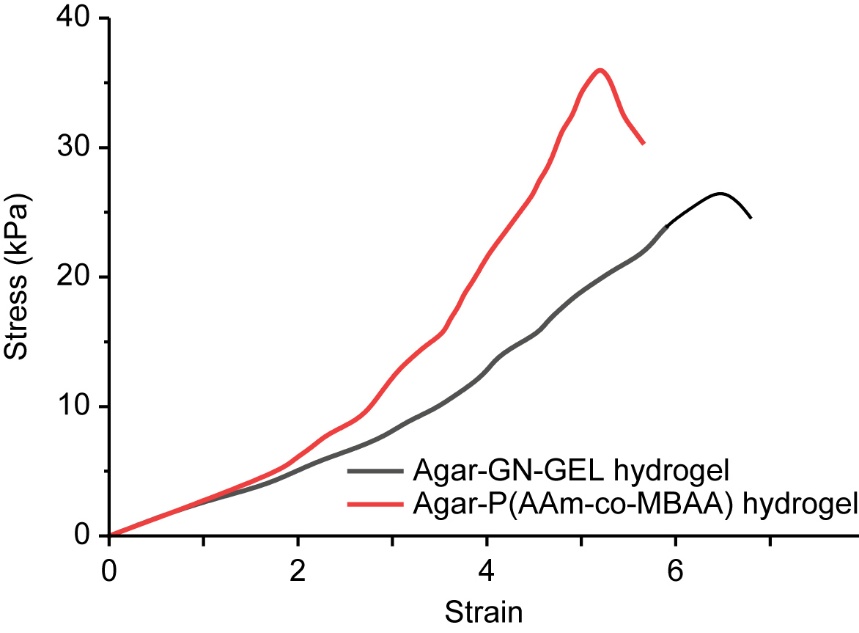
**

**Figure S2.** Stress-strain curves of two hydrogels. A compressive force was applied to the hydrogel samples (1 cm³) separately, measuring the resulting stress and strain. The elastic modulus is calculated from the slope of the initial linear region of the stress-strain curve.


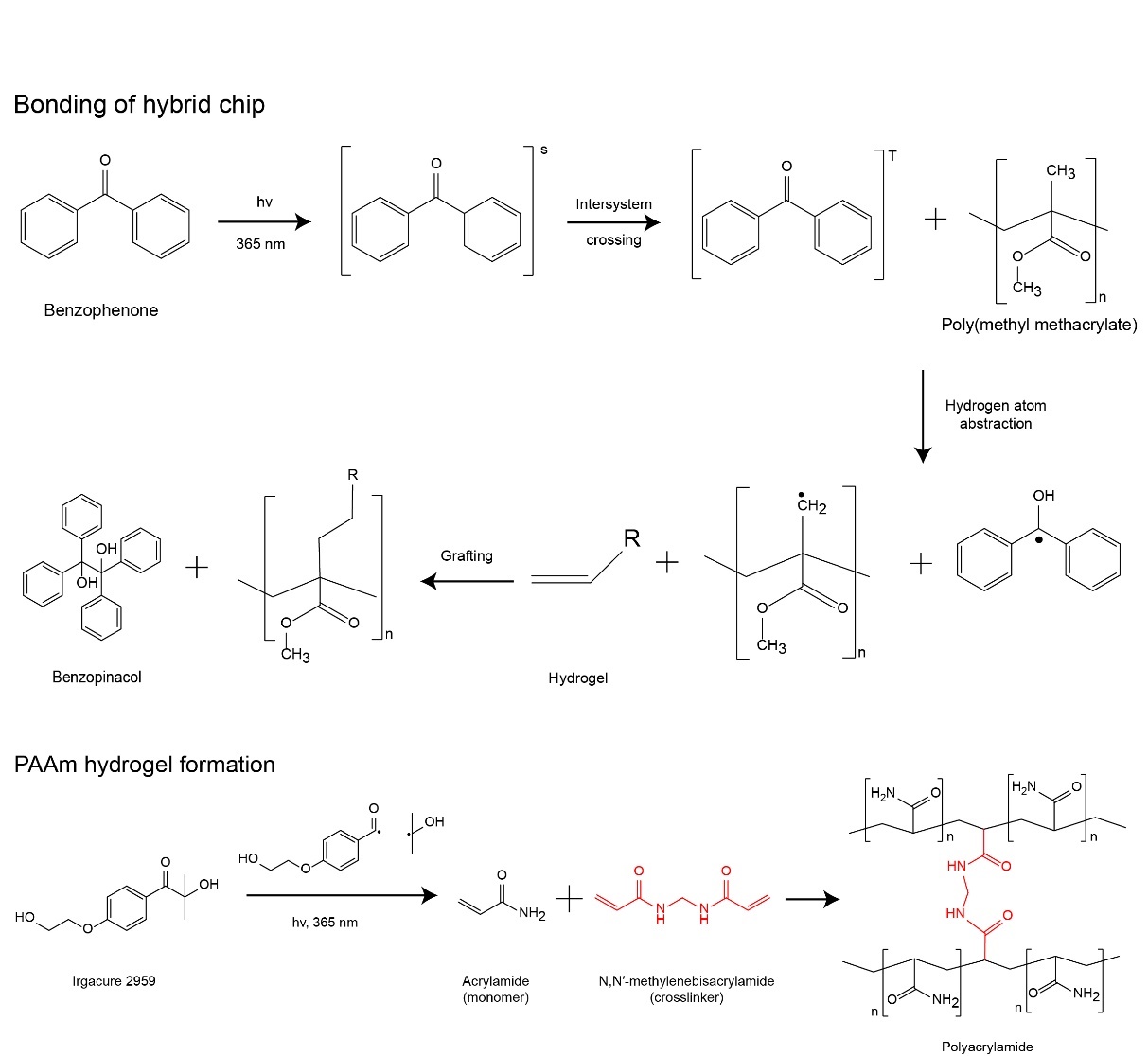


**Figure S3.** P(AAm-co-MBAA) crosslinking and bonding mechanism.


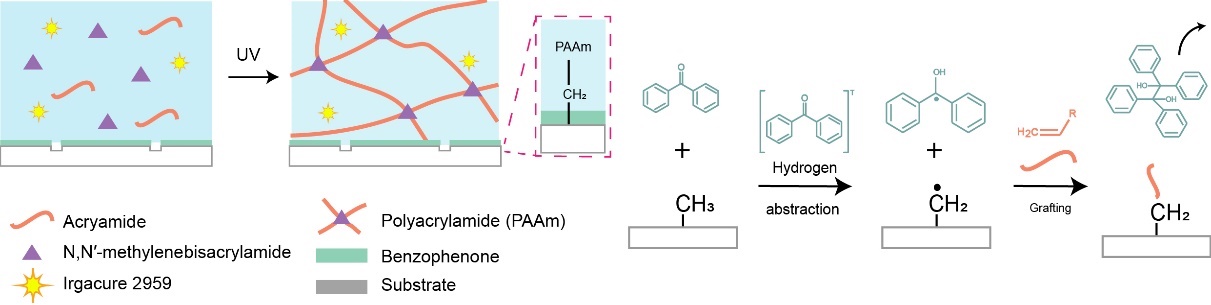


**Figure S4.** Illustration of bonding principle of Agar-P(AAm-co-MBAA) hydrogel on plastic substrate.


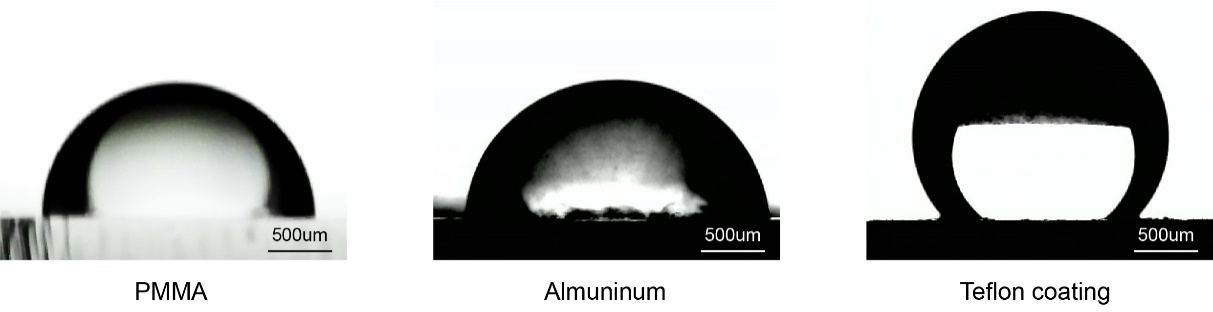


**Figure S5.** Optical images of measuring water contact angles on different materials.

**Table S3.** Contact angle of different materials.

|  | PMMA | Aluminum | Teflon coating |
| --- | --- | --- | --- |
| Contact angle (°) | 89 | 85 | 132 |


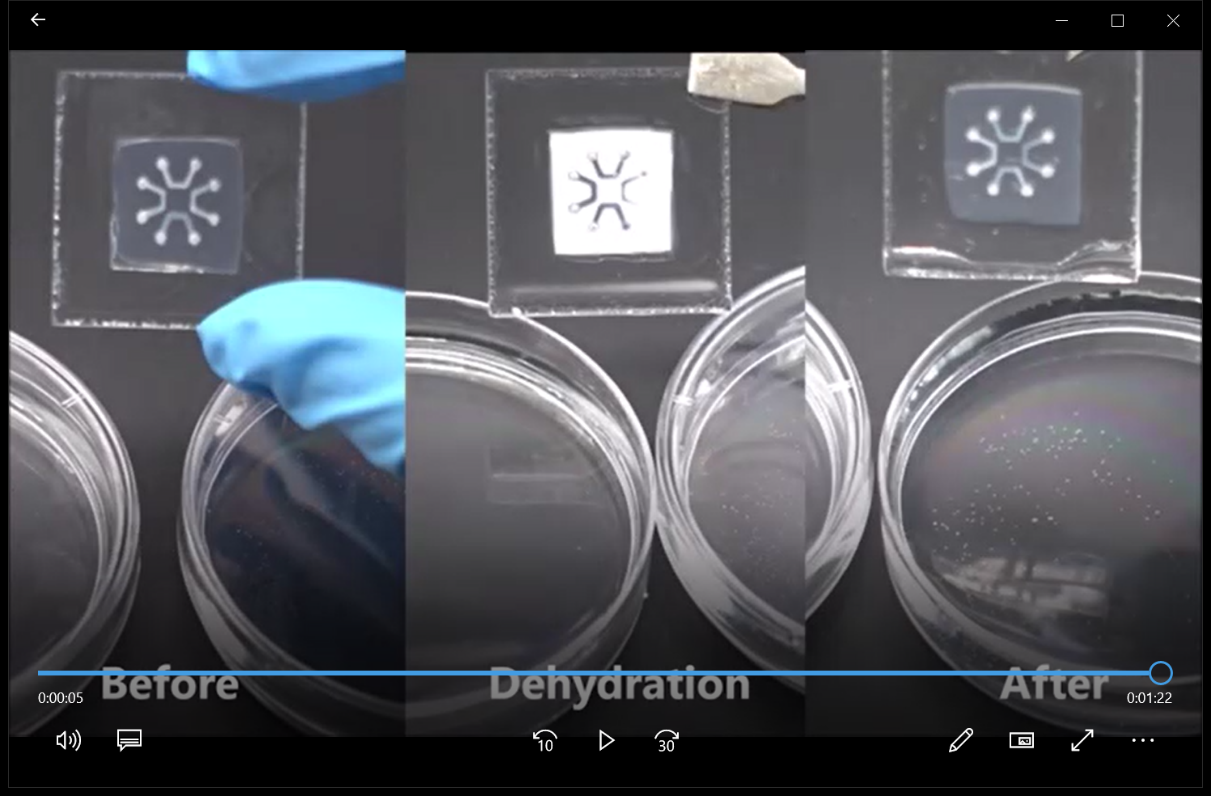


**Figure S6.** Recovery test. The hybrid chip (left) was first immersed into ethanol until the hydrogel changed color from colorless to white for dehydration (middle). Then, the chip was immersed into DI water until the hydrogel changed color from white to colorless for the recovering (right).


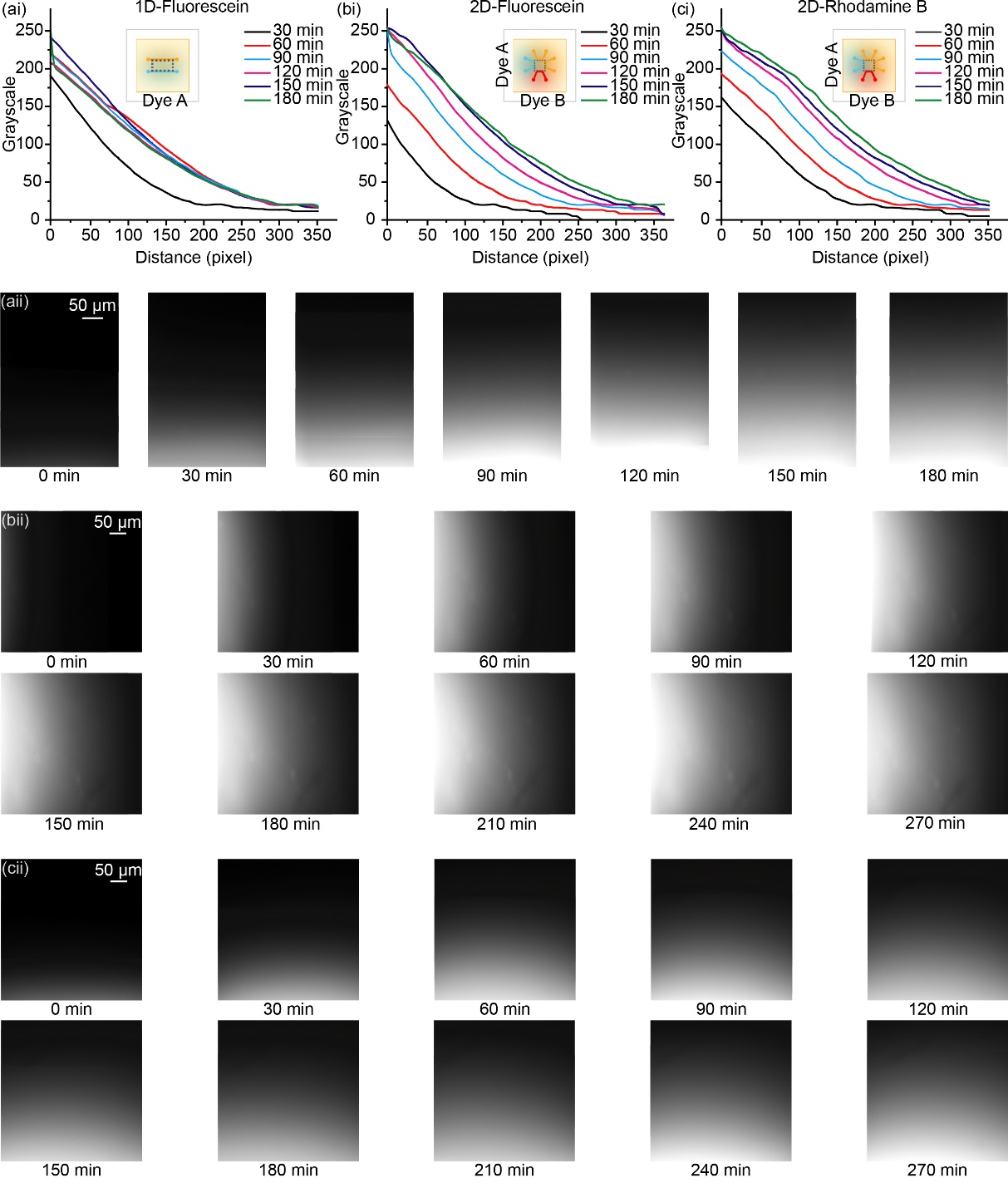


**Figure S7.** Diffusion stability test under fluorescence microscope. (a) For 1D diffusion test, fluorescein (100μg/mL) and D.I water were injected into two parallel channels separately. For 2D diffusion test, fluorescein (100μg/mL) (b) and rhodamine B (100μg/mL) (c) solutions were injected into two neighboring channels; DI water was injected into the other two channels. (i) Fluorescence intensity of the dyes was measured against distance, quantified by gray value after stabilization. (ii) fluorescence images of the chip were recorded every 30 minutes.


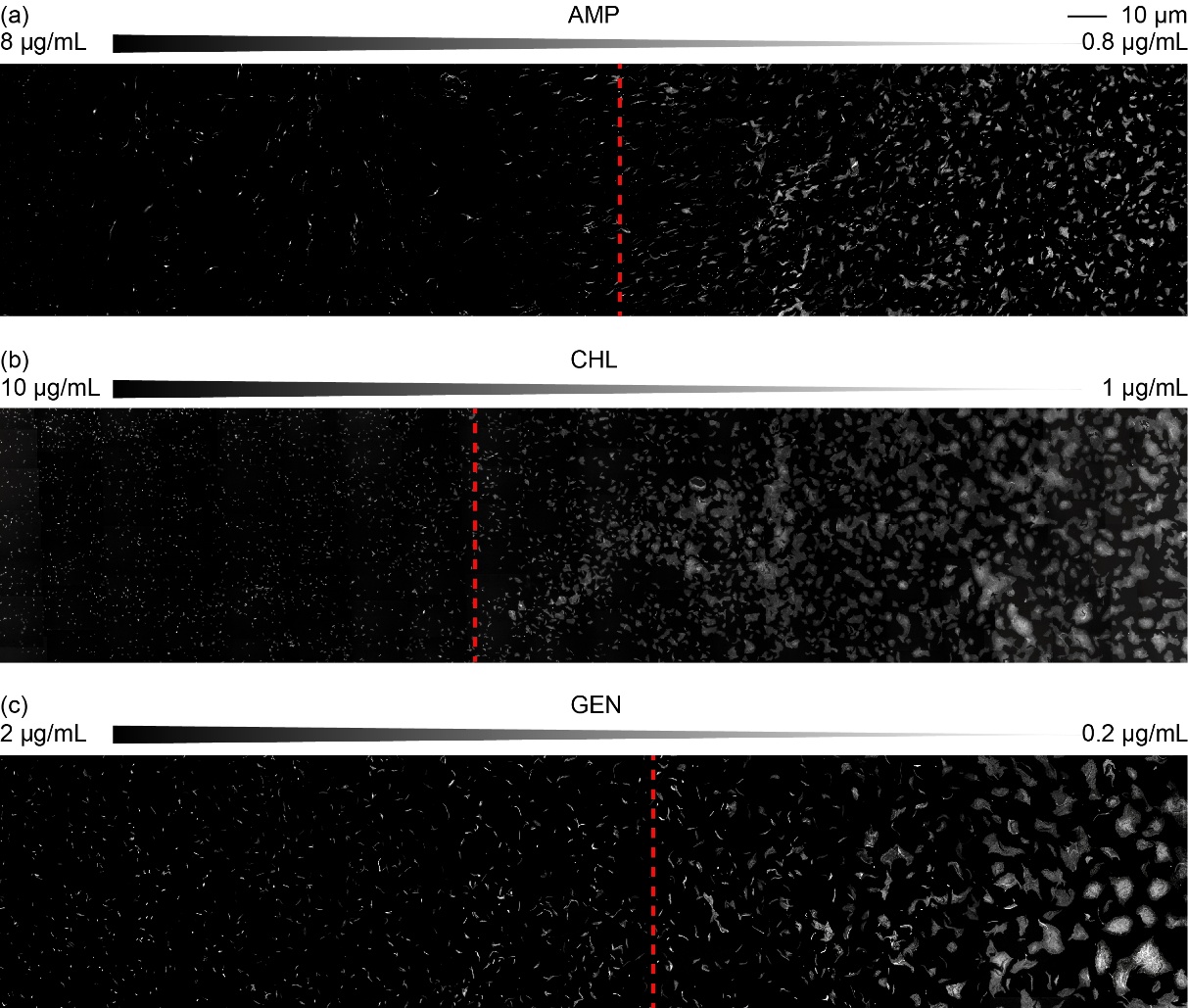


**Figure S8.** 1D AST for GFP-expressed *E. coli*. The drug solution of AMP (a), CHL (b), and GEN (c) were diffused from the left to the right separately. The AST results were recorded under fluorescent microscope after 2 hours incubation. The red dot line is the MIC line.

**
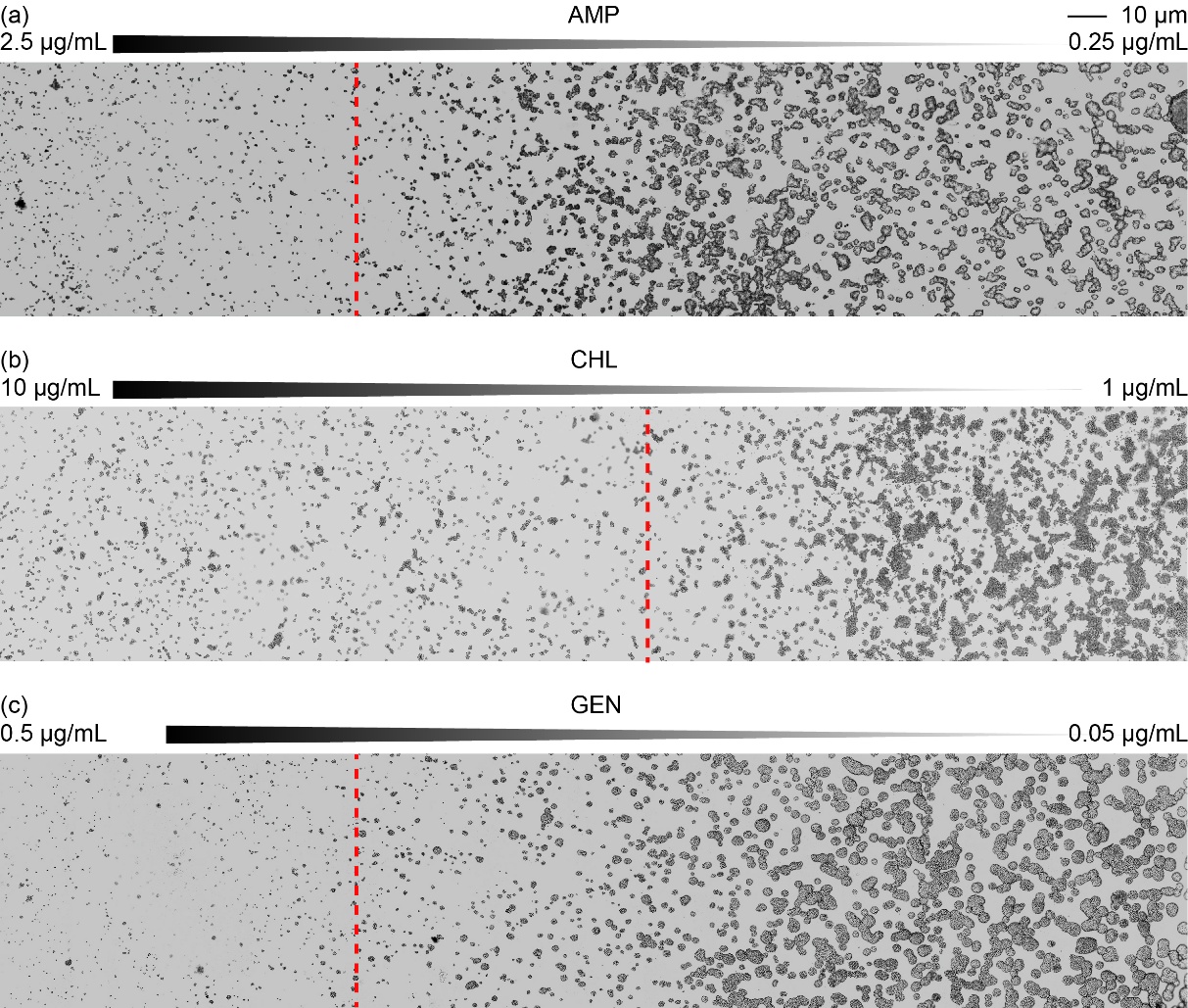
**

**Figure S9.** 1D AST for *S. aureus*. The drug solution of AMP (a), CHL (b), and GEN (c) were diffused from the left to the right separately. The AST results were recorded under an optical microscope after 4 hours incubation. The red dot line is the MIC line.


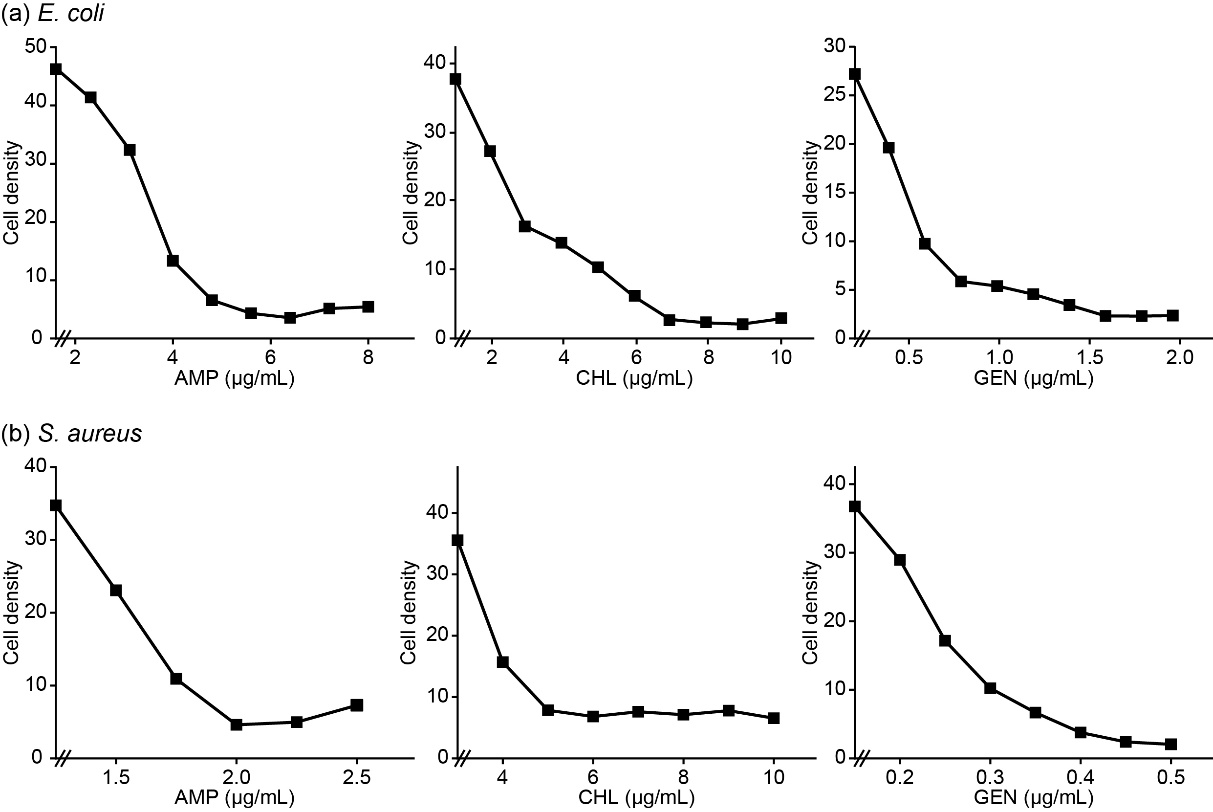


**Figure S10.** Diagram of AST results measured by ImageJ. (a) 1D AST results of GFP-expressed *E. coli*. (b) 1D AST results of *S. aureus*.


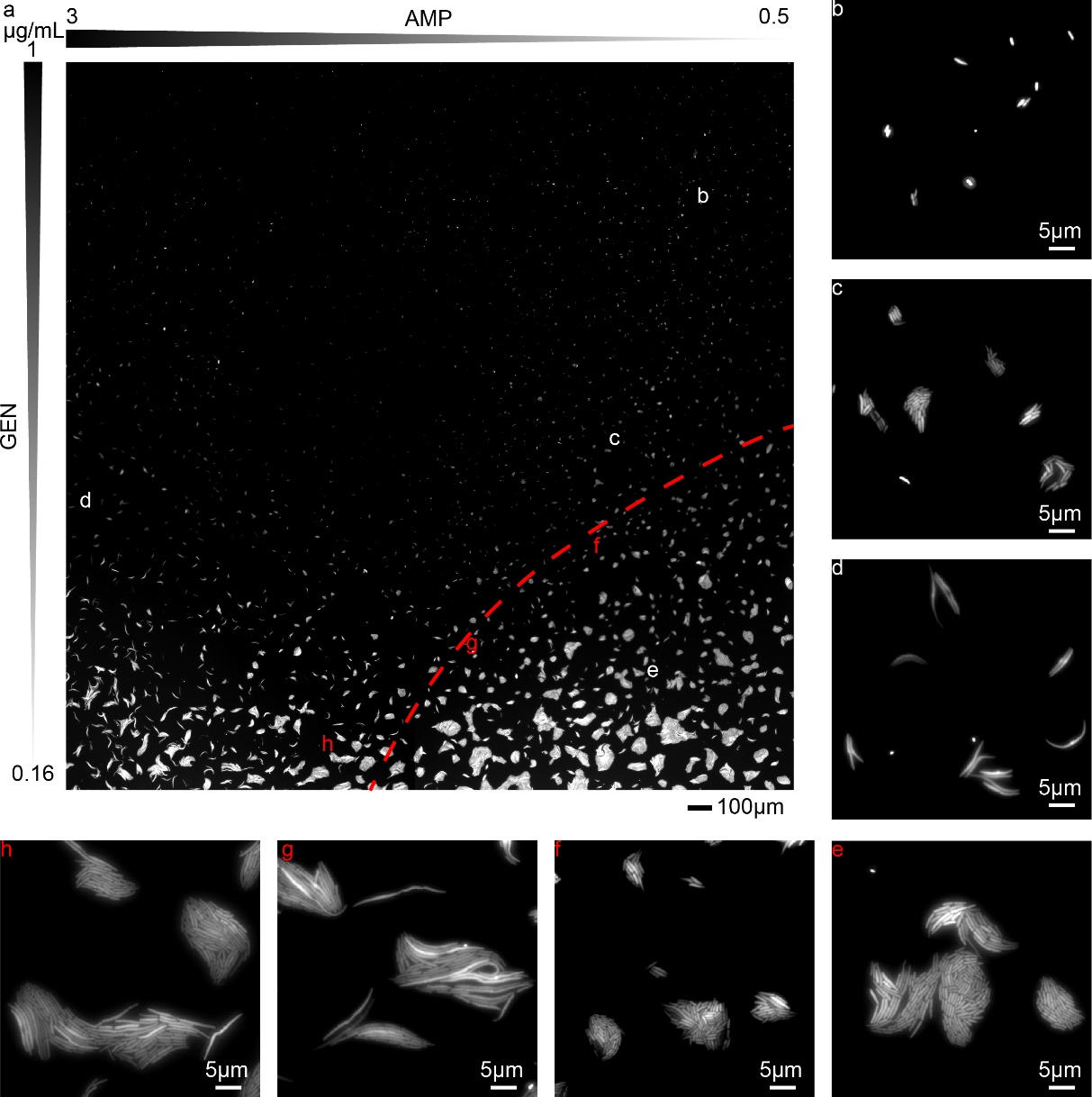


**Figure S11.** 2D AST results of GFP-expressed *E. coli*. (a) 2D AST for combined drug study using GFP E. coli as sample. Ampicillin gradient ran from the left to the right, while gentamicin gradient was generated from the top to the bottom on the device. The red dot line is the MIC line. (b-h) The morphologies of E. coli at different concentrations of two antibiotics.


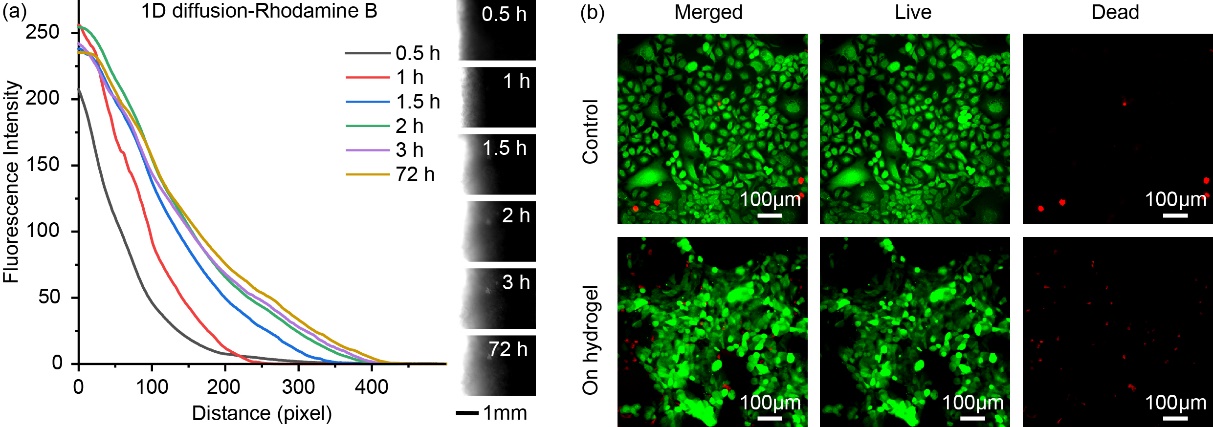


**Figure S12**. An Agar-GN-GEL hydrogel-plastic hybrid chip. (a) The microstructured PMMA was modified through plasma treatment to introduce hydroxyl groups, enhancing adhesion for subsequent steps. Concurrently, approximately 10 µL of colloidal silica solution, which serves as an adhesive, was evenly applied to the Agar-GN-GEL hydrogel surface. The silica nanoparticles in the solution were adsorbed onto the gel. Finally, the Agar-GN-GEL hydrogel was pressed onto the PMMA substrate with a contact pressure of 24 kPa for 3 hours to facilitate adhesion. During this pressing process, the silanol groups on the silica nanoparticles (adsorbed on hydrogel) interacted with the hydroxyl groups on the adjacent PMMA surface, promoting adhesion through hydrogen bonding. (ii) Photo of Agar-GN-GEL hydrogel-plastic hybrid chip. (b) Diffusion stability test under fluorescence microscope. (c) Confocal microscope images of A549 cells after 3 days of culture. The cells grown on a cell culture dish (upper image, control) and those cultured on the Agar-GN-GEL hydrogel (lower image) were stained with fluorescein diacetate (live, green) and propidium iodide (dead, red) to assess cell viability.

**Supplemental Video 1.** Demonstration of our hydrogel-plastic hybrid automatic device. This movie demonstrated the operational process of AST using our automatic device.

**Supplemental Video 2.** Recovery test. This movie includes three parts. 1. Dehydration test. The hybrid chip was dehydrated in ethanol for 25 min. 2. Recovery test. The dehydrated hybrid chip was immersed in DI water for 10 min to recover. The color change of the hydrogel was clearly observed during dehydration (white) and after recovery (colorless).

**References**

[1] K. Výborný, J. Vallová, Z. Kočí, K. Kekulová, K. Jiráková, P. Jendelová, J. Hodan, Š. Kubinová, *Sci Rep* **2019**, *9*, 10674.

[2] W. Liu, K. Han, M. Sun, Z. Huang, J. Wang, *Adv Mater Technol* **2019**, *4*, 1800434.

[3] H. Sun, Z. Liu, C. Hu, K. Ren, *Lab Chip* **2016**, *16*, 3130.

[4] C. Shen, Y. Li, Y. Wang, Q. Meng, *Lab Chip* **2019**, *19*, 3962.

[5] CELLINK, “Stock solutions,” can be found under https://www.cellink.com/product-category/base-materials/stock-solutions/, **2025**.

[6] R. Abbasi, T. B. LeFevre, A. D. Benjamin, I. J. Thornton, J. N. Wilking, *Lab Chip* **2021**, *21*, 2050.

[7] J. Nie, Q. Gao, Y. Wang, J. Zeng, H. Zhao, Y. Sun, J. Shen, H. Ramezani, Z. Fu, Z. Liu, *Small* **2018**, *14*, 1802368.

[8] Y. Zheng, J. Chen, M. Craven, N. W. Choi, S. Totorica, A. Diaz-Santana, P. Kermani, B. Hempstead, C. Fischbach-Teschl, J. A. López, *Proceedings of the national academy of sciences* **2012**, *109*, 9342.

[9] Mayer, “Microfluidic chip fixture customization,” can be found under https://item.taobao.com/item.htm?from=cart&id=542604874989&pisk=gZnZ2_ZGahKwRjfBfXEVLGlL5-ZTkoRSomNbnxD0C5VGflaD3bFCcdE6kol4TjF_5lAT0rD89c9TlSgF3YhEgrpTGSVqGx06d3tSBAEYmjRWV3OZy-XS_NjcjK23B-9QIk9DcvqYmQOVRi494ohGKxnRnw239-ycI-c0xk2zUo2gnSvUt-yRo1c0mpJUe-B, **n.d.**

[10] “Microfluidic chip sampling fixture,” can be found under https://item.taobao.com/item.htm?from=cart&id=674637735498&pisk=gS8E2w_3323EYLwsRdQz_yJOA__d7akXqU65ZQAlO9X3RwsoaKB_PXQI2aJPQLBCAwDdzgAABeGdVTtaaIpwUghdFTXyFQxI1qgX9BQRrLkjlqMeD_VXLk4lKbjGO_c1tNGoPCbRrxMzfzjKuap3sQh0ZlfGB_5ut_vlSNfVgafhtycNI_5YqJvlrflNM_F, **n.d.**

[11] S. Van den Driesche, F. Lucklum, F. Bunge, M. J. Vellekoop, *Micromachines (Basel)* **2018**, *9*, 71.

[12] microfluidic ChipShop, “Male Mini Luer Tube Tuck Connectors for 1/32" OD Tubing,” can be found under https://darwin-microfluidics.com/products/male-mini-luer-tube-tuck-connectors-for-1-32-od-tubing-pack-of-10, **n.d.**

[13] “Luer adapters,” can be found under https://biotechfluidics.com/products/connectors/luer-adapters/, **n.d.**

[14] S. Garcia, R. Sunyer, A. Olivares, J. Noailly, J. Atencia, X. Trepat, *Lab Chip* **2015**, *15*, 2606.

[15] W. Wang, L. Li, M. Ding, G. Luo, Q. Liang, *Biochip J* **2018**, *12*, 93.

[16] S. Bang, S. Na, J. M. Jang, J. Kim, N. L. Jeon, *Adv Healthc Mater* **2016**, *5*, 159.

[17] S. Cosson, M. P. Lutolf, *Sci Rep* **2014**, *4*, 1.

[18] B. P. Mahadik, T. D. Wheeler, L. J. Skertich, P. J. A. Kenis, B. A. C. Harley, *Adv Healthc Mater* **2014**, *3*, 449.

[19] B. Carrion, C. P. Huang, C. M. Ghajar, S. Kachgal, E. Kniazeva, N. L. Jeon, A. J. Putnam, *Biotechnol Bioeng* **2010**, *107*, 1020.

[20] X. Hu, S. Zhao, Z. Luo, Y. Zuo, F. Wang, J. Zhu, L. Chen, D. Yang, Y. Zheng, Y. Zheng, *Lab Chip* **2020**, *20*, 2228.

[21] S. M. Cho, S. Kim, Y. Kim, C. Hwang, T. Kim, S. H. Cheon, J. Y. Kim, C. S. Ah, J. Song, H. Ryu, *Adv Mater Technol* **2019**, *4*, 1800478.

[22] Droplet Genomics, “Cell Barcoding Chip,” can be found under https://darwin-microfluidics.com/products/cell-barcoding-chip-pdms?setCurrencyId=2, **n.d.**

[23] BFlow, “Microvascular and barrier chip,” can be found under https://darwin-microfluidics.com/products/microvascular-and-barrier-chip, **n.d.**

[24] EA., “VasKit,” can be found under https://www.cellink.com/product/vaskit/, **2019**.

[25] TissUse, “HUMIMIC Chip4 - 5-organ-chip,” can be found under https://darwin-microfluidics.com/products/humimic-chip4-5-organ-chip/, **n.d.**

[26] microfluidic ChipShop, “Channel Interaction Chip - Mini Luer,” can be found under https://darwin-microfluidics.com/products/channel-interaction-chip-mini-luer, **n.d.**

[27] MEPSGEN, “MEPS-TBC-WL organ-on-a-chip,” can be found under https://darwin-microfluidics.com/products/meps-tbc-wl-organ-on-a-chip-pack-of-12, **n.d.**

[28] J. Yu, E. Berthier, A. Craig, T. E. de Groot, S. Sparks, P. N. Ingram, D. F. Jarrard, W. Huang, D. J. Beebe, A. B. Theberge, *Nat Biomed Eng* **2019**, *3*, 830.

[29] C. R. Brown, B. Farshchian, P.-C. Chen, T. Park, S. Park, M. C. Murphy, in *ASME International Mechanical Engineering Congress and Exposition*, **2011**, pp. 633–637.

[30] C. R. Brown, X. Zhao, T. Park, P.-C. Chen, B. H. You, D. S. Park, S. A. Soper, A. Baird, M. C. Murphy, *Microsyst Nanoeng* **2021**, *7*, 1.

[31] X. Zhao, D. S.-W. Park, S. A. Soper, M. C. Murphy, *Journal of Microelectromechanical Systems* **2020**, *29*, 894.
